# Single-cell RNA-sequencing of herpes simplex virus 1-infected cells connects NRF2 activation to an antiviral program

**DOI:** 10.1038/s41467-019-12894-z

**Published:** 2019-10-25

**Authors:** Emanuel Wyler, Vedran Franke, Jennifer Menegatti, Christine Kocks, Anastasiya Boltengagen, Samantha Praktiknjo, Barbara Walch-Rückheim, Jens Bosse, Nikolaus Rajewsky, Friedrich Grässer, Altuna Akalin, Markus Landthaler

**Affiliations:** 1 Berlin Institute for Medical Systems Biology, Max-Delbrück-Center for Molecular Medicine in the Helmholtz Association, Robert-Rössle-Strasse 10, 13125 Berlin, Germany; 2 Institute of Virology, Saarland University Medical School, Kirrbergerstrasse Haus, 4766421 Homburg/Saar, Germany; 3 Institute of Virology and Center of Human und Molecular Biology, Saarland University, Saarbrücken, Germany; 4 Heinrich Pette Institute (HPI), Leibniz Institute for Experimental Virology, Hamburg, Germany; 5 IRI Life Sciences, Institute für Biologie, Humboldt Universität zu Berlin, Philippstraße 13, 10115 Berlin, Germany

**Keywords:** Gene regulatory networks, Herpes virus, Virus-host interactions, Transcriptomics

## Abstract

Herpesvirus infection initiates a range of perturbations in the host cell, which remain poorly understood at the level of individual cells. Here, we quantify the transcriptome of single human primary fibroblasts during the first hours of lytic infection with HSV-1. By applying a generalizable analysis scheme, we define a precise temporal order of early viral gene expression and propose a set-wise emergence of viral genes. We identify host cell genes and pathways relevant for infection by combining three different computational approaches: gene and pathway overdispersion analysis, prediction of cell-state transition probabilities, as well as future cell states. One transcriptional program, which correlates with increased resistance to infection, implicates the transcription factor *NRF2*. Consequently, Bardoxolone methyl and Sulforaphane, two known *NRF2* agonists, impair virus production, suggesting that *NRF2* activation restricts viral infection. Our study provides insights into early stages of HSV-1 infection and serves as a general blueprint for the investigation of heterogeneous cell states in virus infection.

## Abstract (web summary)

Herpes simplex virus 1 (HSV-1) induces major reprogramming of the host cell environment. Here, the authors profile the transcriptomes of individual HSV-1-infected human fibroblasts and provide evidence of an antiviral program mediated by the activation of the host transcription factor NRF2.

## Introduction

Herpes simplex virus-1 (HSV-1) is one of nine known herpes viruses that affect humans. Although an estimated 80% of the worldwide population is infected in a quiescent, latent form, the virus may cause a variety of diseases during lytic replication and reactivation^1^. A hallmark of HSV-1 is the way it alters the cellular RNA metabolism and RNA content on many levels. On the one hand, viral mechanisms activate transcription of viral genes and interfere with splicing regulation, RNA polymerase II transcription, and RNA stability to inhibit synthesis of cellular proteins^2–8^. On the other hand, virus entry affects a range of cellular pathways, which may in turn lead to transcriptional activation or repression of downstream target genes^9^.

Viral infection is a dynamic process driven by the interplay of antiviral cellular pathways and viral mechanisms, which evolved to suppress them. Incoming HSV-1 virions bind to receptors on the cell surface, including *NECTIN1*/*NECTIN2*, the *TNF* receptor superfamily member 14 (*TNFRSF14*)^10^, and the members of the integrin family^11^, resulting in activation of cellular pathways such as nuclear factor (NF-κB), or of the transcription factors *IRF3* and *IRF7*^11^. After entry into the host cell, a range of pattern recognition receptors sense viral DNA or RNA, such as the cyclic GMP-AMP synthase *MB21D1* (also known as *cGAS*)^12,13^. Eventually, these pathways can lead to the induction of inflammatory cytokines and interferons^10^.

The characterization of cellular heterogeneity due to the activation of different host pathways and the progression of viral infection is of great interest. Recent single-cell RNA-sequencing (scRNA-seq) efforts provide an unbiased characterization of virus–host interactions in individual cells, which are masked at the population level^14–23^. However, deeper insights into unique molecular signatures and discovery of specific cell subsets can be obtained by increased sequencing depth and the application of advanced analytical approaches to study the course of viral infections.

Here, we profile deep transcriptomes of tens of thousands of individual cells harvested before and at several times post HSV-1 infection. Our results relate the progression of infection to cell cycle phases, and define a precise temporal order of viral gene expression. The depth of data and using unspliced messenger RNA (mRNA) as a predictor for future cell states allows us to connect the course of infection to the activity of specific host cell genes and pathways. Particularly, we investigate the relationship of HSV-1 infection and the transcription factor *NRF2*, which is activated during infection, and demonstrate that the *NRF2* agonists Bardoxolone methyl and dl-sulforaphane impair a productive viral replication. Overall, our study provides insights into early stages of HSV-1 infection, and an analytical framework to study viral infections using scRNA-seq.

## Results

### scRNA-seq of HSV-1-infected primary fibroblasts

**Fig. 1:**
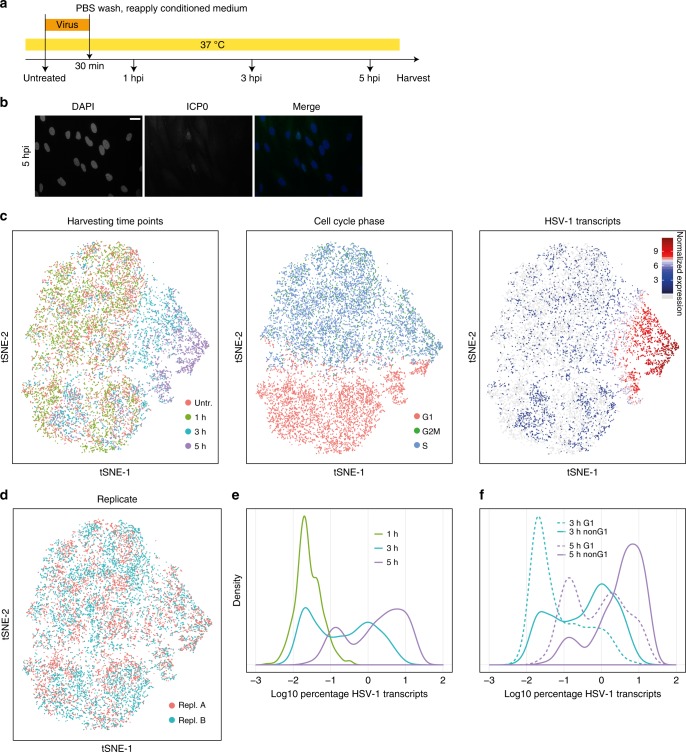
Single-cell RNA-sequencing of HSV-1-infected primary human fibroblasts shows cell cycle dependency. **a** Infection protocol. In parallel to single-cell RNA-sequencing, cells were harvested for bulk mRNA-sequencing and *ICP0* immunofluorescence staining. **b** *ICP0* immunofluorescence staining at 5 hpi. Scale bar: 20 µm. **c** Global display of scRNA-seq data as tSNE maps. Cells were colored by, from left to right, harvesting time points, cell cycle phase, and the normalized values of the sum of HSV-1 transcripts as a marker for the progression of infection. Cells without HSV-1 transcripts are in light gray. **d** tSNE maps with cells colored by replicate. **e** Relative densities of the percentage of viral transcripts (log 10 transformed) per cell for the three time points post infection. **f** Relative densities of the percentage of viral transcripts per cell (log 10 transformed) for G1 and non-G1 cells for cells harvested at 3 and 5 hpi

To investigate the heterogeneity of molecular phenotypes in the first hours of viral infection, we infected primary normal human dermal fibroblasts (NHDFs) with HSV-1 at a multiplicity of infection (MOI) of 10 (Fig. 1a, b) and profiled the transcriptomes of uninfected cells as well as cells harvested at 1, 3, and 5 h post infection using the droplet-based single-cell sequencing (Drop-seq)^24,25^. For further analysis, only cells with more than 2000 detected genes were used, a threshold that has been previously shown to reduce technical variability^26^. An overview of the dataset (Supplementary Table 1), number of characterized cells (Supplementary Table 2), distribution of unique molecular identifiers (nUMIs), that is, the number of individually detected mRNA molecules per cell, and the number of detected genes (nGene) (Supplementary Fig. 1a), as well as correlation between scRNA-seq and bulk RNA-seq (Supplementary Fig. 1b) are provided in the Supplementary information. Low-reproducibility genes (Supplementary Data 1) were subsequently omitted or flagged.

The analyzed cells clustered based on harvesting time point, cell cycle markers, and the amount of viral mRNA, suggesting that the strongest contributors to cellular variability were cell cycle state and the progression of infection (Fig. 1c). However, cells did not separate by biological replicates, indicating that replicates provided comparable and reproducible data (Fig. 1d).

The distribution of the viral gene expression per single cell at the different harvesting time points indicated the progression of infection over time (Fig. 1d). Separating cells based on their cell cycle state (G1 vs. non-G1) showed that, for a given harvesting time point, non-G1 cells generally contain more viral transcripts (Fig. 1e), suggesting that S-, G2-, and M-phase cells are more susceptible to viral infection, and/or that the infection progresses faster in these cells.

Consequently, at 5 h post infection (hpi) we observed that cells bearing high levels of HSV-1 mRNA (8–30%) showed a lower nUMI count (host cell and viral genes together) relative to the number of detected genes (Supplementary Fig. 1c), indicating less complex transcriptomes due to a large number of viral transcripts and/or reduction of host cell mRNAs likely as a consequence of the beginning host cell shutoff^2^.

Of note, we detected little or no mRNA of the immediate early genes *RL2* (coding for ICP0) and *RS1* (coding for ICP4), both in bulk RNA-seq and scRNA-seq, which was already observed in a previous study^27^, likely being explained by the unusually high GC content of these mRNAs.

### Stepwise progression of viral gene expression

Within lytic infection, viral genes have been classified as immediate early (α), early (β), and late (γ, γ1, γ2)^1,27^. Since the temporal order of genes expressed from the virus genome is intrinsically encoded in their single-cell expression profiles, we developed an approach to refine the viral gene expression cascade.

**Fig. 2:**
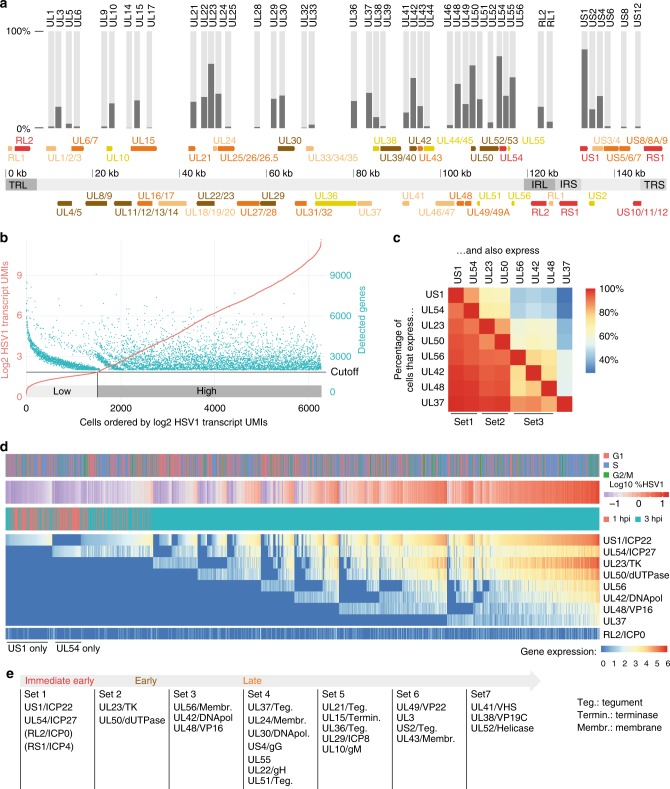
Onset of viral gene expression shows a set-wise emergence of viral transcripts. **a** Arrangement of viral genes on the HSV-1 genome. Immediate early genes were shown in red, early genes in brown, and late genes (gamma) in orange, γ1 in light brown, and γ2 in dark yellow. Genome segments were shown as light gray (unique regions) and dark gray areas (IRL, IRS: large/small internal repeats; TRL, TRS: large/small terminal repeats). Overlapping genes were merged. The bar plot on top shows in which percentage of “HSV-1 high” cells (see **b** for definition) the respective gene was detected. **b** Relationship between normalized levels of HSV-1 transcripts and the number of detected genes (human and viral) shows two categories of cells. Cells with at least one viral transcript were sorted by normalized HSV-1 transcript UMIs. Each cell is repesented by a red dot (log(2)-transformed sum of HSV-1 transcript UMIs, left axis) and a green dot (number of genes detected, right axis). Note that only cells with more than 2000 detected genes were used. The horizontal black line denotes the cutoff between high and low. For subsequent analysis, only the 3896 “high HSV-1” cells were used, in order to reduce the sampling error caused by the detection rate. **c** Percentages indicating co-occurrences of genes. Read as following, for example: 85% of cells that have *US1* also have *UL54* (top row, second field from left, dark orange) but only 29% have *UL37* (top row, last field, dark blue), whereas 99% of cells that have *UL37* also have *US1* (bottom row, first field from left, red). **d** Heatmap of expression values of the first eight expressed viral genes in “HSV-1 high” cells, harvested at 1 and 3 hpi. Rows (genes) and columns (cells) were sorted as described in the main text. Above the heatmap, cell cycle, harvesting time point, and log 10-transformed percentage of viral transcripts. For the latter, blue colors indicate values in the first and second peak of the bimodal distribution from Fig. 1e, respectively. **e** Proposition for a refined scheme of temporal categorization of viral genes

As an initial proxy for the sequential emergence of virus-encoded transcripts, we counted, for each gene, the percentage of infected cells in which it was detected (Fig. 2a, Supplementary Data 2). Interestingly, viral gene transcription appears to start in regions flanking the internal repeat regions, and around gene *UL23*. To reduce the effects of the sampling error (mRNA capture rate) when analyzing individual viral transcripts, we used only cells where the amount of detected viral transcripts was not dependent on the sequencing depth (“high” cells in Fig. 2b).

First, we focused on early viral transcriptional events, and explored cells harvested at 1 and 3 hpi. For the first eight viral genes as defined by the ordering in Fig. 2a, we calculated for each gene pair the percentage of cells that have both genes present. Clustering the gene pairs by co-occurrence frequencies showed a grouping of viral genes (Fig. 2c), such as *UL23* together with *UL50* or a set with *UL56*/*UL42*/*UL49*. For a temporal representation of viral gene expression, cells were first sorted according to the emergence of the eight genes (Fig. 2d), and then by the abundance of *US1* (coding for ICP22), or, if absent, *UL54* (ICP27). Under the assumption that the progression of infection, at least in an early phase, is correlating with the accumulation of viral transcripts, this represents a pseudo-time course of the lytic infection. In agreement with previous findings^1,27^, *US1* and *UL54* were the first genes to be detected (Fig. 2d). Particularly, cells harvested at 1 hpi mostly contained one of these two transcripts (Fig. 2d). Interestingly, a considerable number of cells had only one of the two transcripts present (Fig. 2b, left most part), indicating that at the very beginning of viral transcription, either only *US1* or only *UL54* is turned on. The immediate early gene *RL2* (coding for ICP0) is displayed separately, since its mRNA transcript might be difficult to detect.

Next, we connected the bimodal distribution of viral transcripts per single cell (Fig. 1e) to the sequential expression of viral genes in two ways. First, cells in the first peak (Fig. 2d) contain mainly transcripts from one or two viral transcripts. Second, we generated smoothened two-dimensional densities of cells by plotting the number of detected genes vs. the percentage of viral transcript per cell (Supplementary Fig. 2a). Together, this indicates that the bimodality in Fig. 1e arises from the transition between *US1*/*UL54* expression and later stages of infection.

To include viral genes that are transcribed later, we analyzed cells harvested at 5 hpi the same way as the 1/3 hpi cells in Fig. 2c, d (Supplementary Fig. 2b, c). Again, the clustering revealed a set-wise emergence of viral genes, that is, sets of genes being transcribed together.

In order to extend the analysis on the entire transcriptome of the cells, including host cell genes, we calculated the likelihood of transitions between single cells based on diffusion maps^28,29^. Cells were then clustered by these transition probabilities (Supplementary Fig. 2d for 1/3 hpi and Supplementary Fig. 2e for 3/5 hpi). Here, discrete clusters of cells could be observed. These clusters obviously have high transition probabilities within themselves. However, they are also connected by high transition probabilities to specific other clusters, indicating that the infection on the transcriptional level progresses in a stepwise manner rather than a continuum. Importantly, the set-wise emergence seen in Fig. 2c and Supplementary Fig. 2b is recapitulated here by discrete and set-wise transitions of the occurrences of viral genes per cluster.

Decades of work have classified viral genes into immediate early, early, and late (γ, γ1, γ2)^1^. Based on the single-cell data, we now propose, as a refinement, a set-wise emergence of viral genes as shown in Fig. 2e.

### Infection-induced *RASD1*/*RRAD* genes reduce virion production

**Fig. 3:**
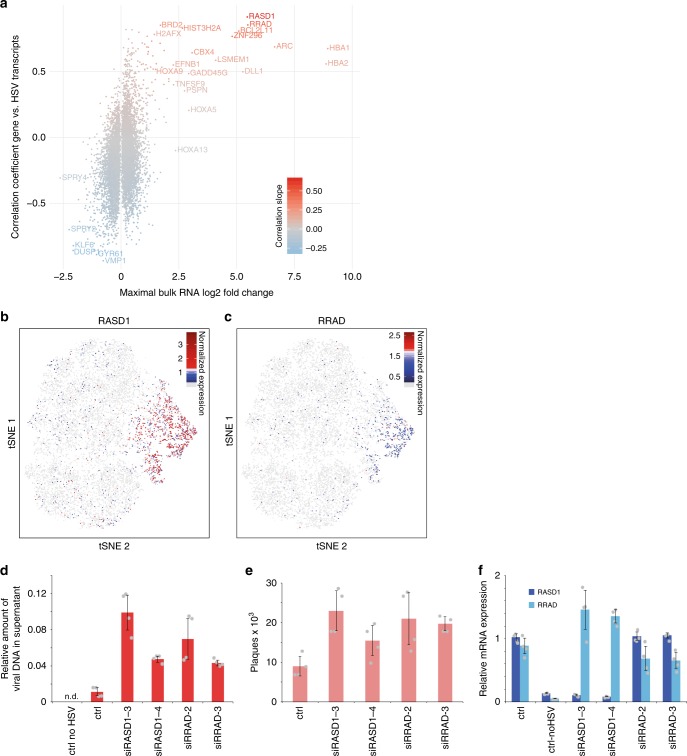
Correlating host cell and viral gene expression reveals candidates modulating infection. **a** Relationship between differentially expressed genes and viral transcription. The horizontal axis shows the maximal log(2)-transformed bulk RNA-seq fold change of the three time points after infection compared to uninfected cells, the vertical axis, and the linear regression coefficient of the gene expression with the sum of viral transcripts in bins of 20 cells, using only high HSV-1 cells as defined in Supplementary Fig. 2a. Color represents the slope of the linear regression. Source data for this panel is provided in Supplementary Data 3. **b**, **c** Distribution of normalized expression of *RASD1* (**b**) and *RRAD* (**c**) on the tSNE projection introduced in Fig. 1. Cells without detectable expression are colored in light gray. **d**, **e** RNAi of HSV-1 transcription-dependent factors *RASD1* and *RRAD*. Viral DNA in the cell culture supernatant was measured using plaques assays (**d**) or qPCR (**e**) (quantified using serial dilutions of a virus stock with known activity, a value of 1 corresponding to 10^6^ PFU/ml). Bar plots indicate means, error bars denote standard deviations, and the individual measurement values (two each from *n* = 2 biologically independent samples) are shown as gray dots. **f** RT-qPCR of RNAi samples. *RASD1* and *RRAD* mRNA levels were normalized using *GAPDH* mRNA values and to control cells. The individual measurement values are shown as gray dots. Source data for the bar plots are provided in the Source Data file

To study changes in viral gene expression upon infection, we first examined differential gene expression by bulk mRNA-seq (Supplementary Fig. 3a). To identify genes differentially expressed only in infected cells, we plotted the correlation of host gene expression to viral transcripts in single cells against the maximal fold change in the population mRNA-seq data (Fig. 3a, Supplementary Data 3).

Among host genes, activated only in infected cells, we identified hemoglobin α genes, *HBA1* and *HBA2* (Fig. 3a), which were previously shown to be induced by the viral transcription factor ICP4^7^. Additionally, two genes encoding Ras-related small GTPases, *RASD1* (Fig. 3b) and *RRAD* (Fig. 3c), showed strong positive correlations with viral transcripts. Similarly, we found that *RASD1* and *RRAD* were up-regulated in previously published microarray, RNA-seq, and ribosome profiling datasets^5,6,30,31^, indicating that the induction of these two genes is independent of the cell type and virus strain. To investigate the influence of *RASD1* and *RRAD* on viral production, we depleted *RASD1* and *RRAD* by small interfering RNA (siRNA) knockdown prior to infection (Fig. 3c–e), and observed increased virion production at 16 hpi, suggesting that both genes posses some antiviral activity.

### Subpopulation of cells defined by the expression of marker genes

**Fig. 4:**
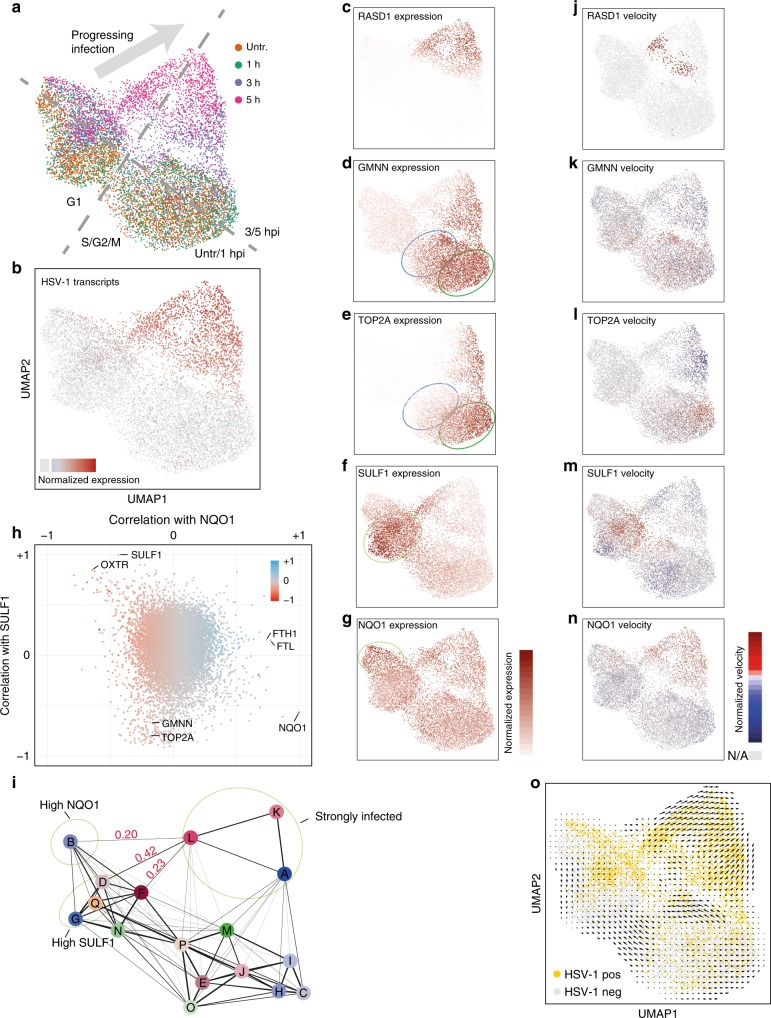
Marker genes define subpopulations with different transition probabilities into infection. **a** Cells were projected on a two-dimensional map using UMAP based on gene expression values, with cells colored by harvesting time point. Main distinguishing features were marked in gray. **b** Cells colored by amount of HSV-1 transcripts. Cells without HSV-1 transcripts are colored in light gray. **c**–**g** Cells colored by expression values of the indicated genes. Color scales are shown to the right of these panels. Areas containing cells with relatively high expression levels were marked with a light green ellipse. The dark green and blue ellipse denote S and G2/M phase cells, respectively. **h** Correlation of gene expression with *NQO1* expression (horizontal axis) and *SULF1* expression (vertical axis) in cells harvested at 3 hpi. Every dot represents a gene, colored by the slope of the linear correlation with *NQO1*. Selected genes were labeled by name. To calculate correlations, only cells with relative normalized expression above a certain threshold were used as for Fig. 2 and viral transcripts (Supplementary Fig. 4c). **i** Groups of cells according to PAGA were labeled A to Q. The line thickness of the connections (graph edges) between the cell clusters (graph nodes) indicate the likelihood that cells can move from one cluster to the other (right). Probabilities (arbitrary scale) are noted in red. Areas of interest are marked with light green ellipses. **j**–**n** Cells colored by RNA velocity values of the indicated genes. Cells for which no value could be calculated were colored in light gray. **o** RNA velocity arrows projected on the UMAP. Cells containing at least one detected viral transcript were colored in light orange, and all others in light gray

To identify distinct subpopulations of cells, defined by a particularly high or low expression of specific marker genes, we used an overdispersion analysis. Overdispersion analysis selects genes that have variance of mRNA expression, higher than expected, for their average level of expression. Such high variance indicates that these genes are highly expressed in only a subset of cells in the population. Not surprisingly, cell cycle, viral genes, and host cell genes correlating with viral infection, such as *RASD1*, appeared as the strongest gene expression markers (Fig. 4a–e). In addition, two other gene sets, not directly related to the progression of infection and the cell cycle, defined distinct subpopulations. Importantly, the genes discussed here do not necessarily by themselves influence the infection, but rather are indicators of a specific cellular state that could favor or impair the infection.

The first subpopulation was marked by high mRNA levels of the sulfatase *SULF1* (Fig. 4f), and the oxytocin receptor *OXTR* (Supplementary Fig. 4a). The second set was characterized by high mRNA levels of the NAD(P)H quinone dehydrogenase 1 (*NQO1*) (Fig. 4g) and the ferritin heavy chain 1 (*FTH1*) (Supplementary Fig. 4b), as well as the ferritin light chain (*FTL*) (not shown) and sequestosome 1 (*SQSTM1*) (not shown).

Whereas *SULF1* was previously shown to be induced by tumor necrosis factor-α (TNFα) in MRC-5 fibroblasts^32^, we found no clear activators for *OXTR*, but indications that pro-inflammatory cytokines, such as IL-1β, IL-6, and TNFα might induce its transcription^33^. For the activation of *NQO1* and the correlating genes *FTH1*, *FTL*, and *SQSTM1*, several reports pointed to the transcription factor *NFE2L2* (also known as *NRF2*)^34–36^.

Pairwise gene expression analysis revealed a strong anti-correlation of *SULF1* and *NQO1* expression in single cells, whereas *FTH1* and *FTL* correlated well with *NQO1* (Fig. 4h). On the other hand, *OXTR* expression levels correlated with *SULF1* but not with *NQO1*, indicating the existence of subpopulations of cells with distinct marker gene expression.

Next, we related these subpopulations to the progression of infection. To this end, we used graph abstraction (PAGA^37^), which calculates transition probabilities between groups of cells (Fig. 4i, Supplementary Fig. 4d). Cells with high *NQO1* levels (group B in Fig. 4i), and therefore high preceding *NRF2* activity, had a relatively low probability to progress further into the infection, compared to cells from groups D and F in Fig. 4i.

### RNA velocity shows transcription bursts and cell cycle arrest

To infer expression dynamics in individual cells, we used RNA velocity^38^. RNA velocity uses sequencing reads originating from introns to measure the amount of nascent mRNA being produced from a certain locus. The ratios of nascent mRNA to mature mRNA transcripts, across multiple cells, can be used to derive transcriptional rates. Changes in transcriptional rates, in multiple effector genes belonging to the same signaling pathway, are an indicator of the pathway’s activity.

RNA velocity values and the number of nascent reads for two genes induced by the infection, *RASD1* and *HOXA9* (Fig. 3), are shown in Fig. 4c, j, and Supplementary Fig. 4d–g. We observed that these two genes were transcriptionally induced in different subgroups of cells, and that the apparent transcriptional shutoff of *RASD1* does not necessarily reflect a general shutoff with the progressing infection, since *HOXA9* transcription is observed in cells in later stages of infection (compare Supplementary Fig. 4d, f). The transcriptional bursts for *RASD1* and *HOXA9* are also reflected in the clusters shown in Supplementary Fig. 2e, where they co-occur with the emergence of specific sets of viral transcripts.

RNA velocity values are shown for the genes mentioned in the previous section are shown in Fig. 4k–n and Supplementary Fig. 4a, b. Cells in the G1 phase that show high *GMNN* RNA velocity (Fig. 4k) are therefore cells that are about to enter the S phase. Interestingly, cells in a more progressive state of infection had low RNA velocity values for both *GMNN* and *TOP2A* (Fig. 4k, l, top part of the maps), which reflects the interruption of the cell cycle by the virus^39^. In addition, RNA velocity allows the prediction of the future state of individual cells on a timescale of hours. The progression of infection clearly emerged as the predominant transition (Fig. 4o), confirming the validity of the approach. Since viral genes barely have introns, the directionality of infection progression is driven by virus-induced host cell genes such as *RASD1* (Fig. 4c, j).

### The transcription factor NRF2 is activated upon infection

**Fig. 5:**
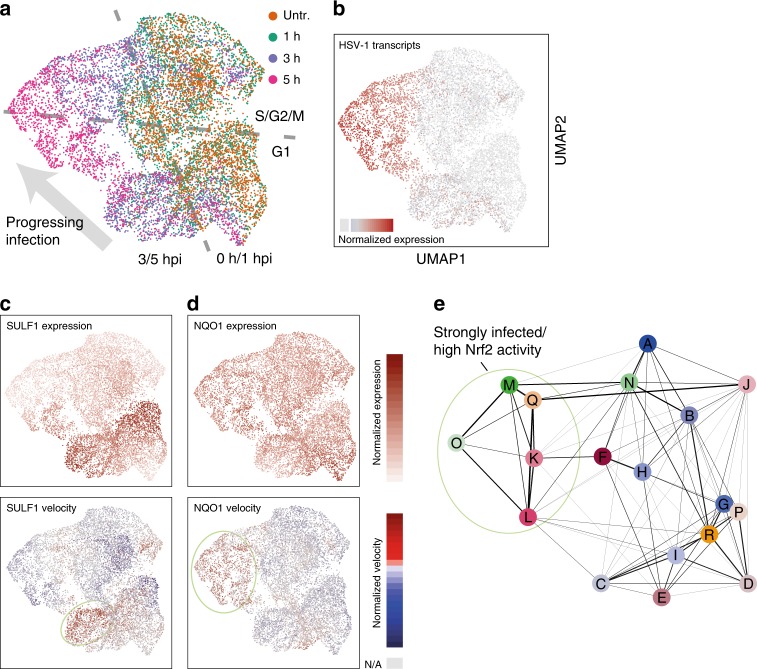
Subpopulations with common transcriptional activity defined by RNA velocity. **a** Cells were projected on a two-dimensional map using UMAP according to RNA velocity values, with cells colored by harvesting time point. Main distinguishing features were marked in gray. **b** Coloring by amount of HSV-1 transcripts. **c** and **d** Cells colored by expression values (top panels) and RNA velocity values (bottom panels) of the indicated genes. Color scales are shown to the right of these panels. For expression values, areas containing cells with relatively high expression levels were marked with a light green ellipse. For RNA velocity, cells for which no value could be calculated were colored in gray. **e** Groups of cells according to PAGA were labeled A to R. Transition probabilities between clusters are proportional to the line thickness between the clusters (right)

We used the inferred RNA velocity (transcriptional rates) as a precise read out of activation of upstream signaling pathways. To visualize pathway activity, we mapped the cells on a two-dimensional embedding not based on the mature mRNA expression levels as in Fig. 4, but based on the RNA velocity values (Fig. 5a, b). Cells with larger amounts of viral transcripts showed relatively high *NRF2* activity as deduced from high *NQO1* RNA velocity (Fig. 5d, lower panel) suggesting that *NRF2* is activated as a part of the cellular defense against the progressing infection. We again applied the PAGA^37^ algorithm to cells clustered based on transcriptional activity (Fig. 5e). The highest probability to proceed into infection are cells in the S/G2/M phases (groups F, N, J), supporting again that these phases of the cell cycles favor infection.

To summarize, we made two observations regarding *NRF2* activity and HSV-1 infection. The analysis of mature mRNA distribution showed that cells with a high level of transcripts of *NRF2*-driven genes, and therefore high preceding *NRF2* activity, have a low transition probability into later stages of infection (Fig. 4). Looking at RNA transcription however showed that cells at later stages of infection appear to respond by increasing transcription of *NRF2* target genes (Fig. 5), which could reflect a cellular defense mechanism against HSV-1 infection.

In order to strengthen our interpretation of the data, we also analyzed an independent experiment with again two biological replicates, where a procedure for synchronized infection at 4 °C^40^ was applied (Supplementary Fig. 5a, Supplementary Table 1). Whereas overall the infection progressed less (compare Supplementary Fig. 5b with Fig. 1e), key aspects of our analysis were reproducible. This included the cell cycle dependency (Supplementary Fig. 5c), the viral gene expression cascade (Supplementary Fig. 5d) and the induction of *RASD1*/*RRAD* (Supplementary Fig. 5e). Due to the weaker infection, there are less infected cells as a separated part on the two-dimensional projection (compare Supplementary Fig. 5f, g with Fig. 4a, b). Still, the anti-correlation of *NQO1*/*SULF1* and the low transition probability of cells with high *NQO1* levels into infection was observed (Supplementary Fig. 5f–j).

### Activated NRF2 restricts HSV-1 infection

Our analysis suggested that NRF2 activation occurs in a subset of infected cells. Under physiological conditions, NRF2 is repressed by KEAP1, which sequesters NRF2 and facilitates its ubiquitination and degradation^41^. Upon disruption of this interaction in response to oxidative stress, NRF2 translocates into the nucleus, and induces transcription of a number of target genes, including *NQO1*. Using the *NRF2* agonist bardoxolone methyl^42^ at final concentrations of 0.1–0.4 µM, we observed an increase in *NQO1* mRNA expression in primary fibroblasts (Supplementary Fig. 6a, left panel) and in HEK 293 cells (Supplementary Fig. 6b), as expected for *NRF2* activation^43,44^. In addition, we used a second *NRF2* agonist, sulforaphane, which induced *NQO1* expression at the previously reported low micromolar range^45^ (Supplementary Fig. 6a). At the concentrations applied here, bardoxolone methyl did not have any apparent effect on cell growth (data not shown). Since, as described above, the progression of infection depends on the cell cycle, we also probed mRNA levels for the cell cycle markers *GMNN* and *TOP2A* introduced in the previous sections (Supplementary Fig. 6a, b, right panels). In the primary fibroblasts but not HEK293 cells, the levels of these mRNAs were somewhat reduced, indicating that bardoxolone methyl could also dampen cell cycle progression or promote cell cycle exit. An experiment with bardoxolone methyl from a different source is shown in Supplementary Fig. 6c.

**Fig. 6:**
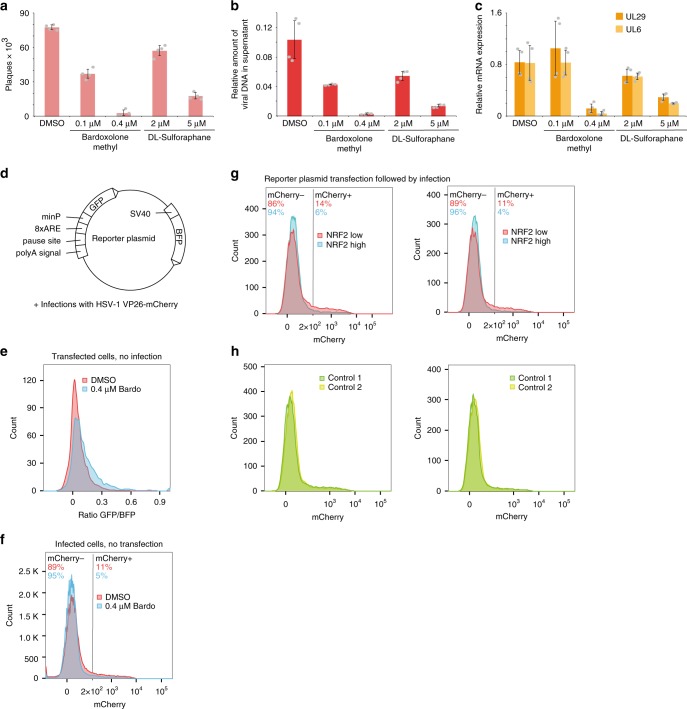
*NRF2* activity counteracts HSV-1 infection. **a**–**c** NHDF cells were infected with HSV-1 at an MOI of 1. After removal of virus inoculum and washing with PBS, conditioned medium supplied with solvent or different concentrations of *NRF2* agonists were added. At 16 hpi, virus production was assessed using plaque assays (**a**), by probing viral DNA in the supernatant using qPCR (**b**), and by measuring viral mRNAs in the RNA isolated from the cells using RT-qPCR (**c**). For all panels, bar plots indicate means, and error bars denote standard deviations; the individual measurement values (two each from *n* = 2 biologically independent samples) are shown as grey dots. Source data are provided in the Source Data file. **d** Design of the *NRF2* reporter plasmid. SV40: simian virus 40 enhancer and promoter; *BFP*: blue fluorescent protein; polyA signal/pause site: polyadenylation signal and pause site for RNA polymerase II; 8×ARE: eight times antioxidant response element; minP: minimal promoter, *GFP*: green fluorescent proteins. **e** HEK 293 cells transfected with the reporter plasmid, and 24 h later treated with DMSO (red) or bardoxolone methyl (blue). Ratios of *GFP* to *BFP* signal were measured by FACS 15 h later and are displayed as overlapping histograms. **f** HEK 293 cells were infected with the HSV-1 *VP26-mCherry* virus at an MOI of 1. After removal of virus inoculum and washing with PBS, conditioned medium supplied with solvent or bardoxolone methyl was added. **g** HEK 293 cells were transfected with the reporter plasmid and 24 h later infected with the HSV-1 *VP26-mCherry* virus at an MOI of 1. At 16 hpi, intensities for *BFP*, *GFP*, and *mCherry* were measured by FACS and the distribution of the *mCherry* signal (which reflects progression of infection) in the *NRF2* low and high populations plotted as histograms. Shown are two representative experiments. **h** as for **g** but with the distribution of the *mCherry* signal in the two control populations as defined in Supplementary Fig. 6f

Next, we tested whether the two *NRF2* agonists modulate HSV-1 infection. To this end, cells were infected and bardoxolone methyl or sulforaphane were added right after removal of the virus inoculum. Both compounds reduced the levels of produced virions at 16 hpi, as measured by plaque assays (Fig. 6a), the amount of viral DNA in the supernatant (Fig. 6b, left panel). The mRNA of the early UL29 and particularly the late *UL6* gene (Fig. 6c) was also reduced, suggesting that late stages of viral transcription/replication and/or virion production are impaired in cells treated with *NRF2* agonists. Similar effects were observed in treated HEK 293 cells (Supplementary Fig. 6e).

To support the hypothesis that high NRF2 activity counteracts the progression of HSV-1 infection, we performed a fluorescence-activated cell sorting (FACS)-based assay using a reporter plasmid to monitor NRF2 activity in HSV-1-infected cells. This reporter plasmid (Fig. 6d) encodes green fluorescent protein (*GFP*), which is preceded by eight antioxidant response elements GTGACNNNGCANNN^46^, known as NRF2-binding sequences, followed by a minimal promoter (see Supplementary File 1 for the sequence). A constitutively expressed BFP controls for transfection efficiency. As expected, transfection of the reporter plasmid into bardoxolone methyl-treated HEK 293 cells increased GFP to BFP ratios when compared to solvent-treated cells (Fig. 6e, Supplementary Fig. 6g), indicating that the reporter plasmid can be used to monitor NRF2 transcriptional activity. The separation of subpopulations and the gating strategy is shown in Supplementary Fig. 6f.

To monitor the progression of HSV-1 infection, we used a HSV-1 virus with *mCherry* fused to the late *VP26* gene, encoding the small capsid protein^47^. Higher mCherry expression per cell indicates a more productive infection. As shown before, the VP26-mCherry virus showed impaired infection upon treatment with bardoxolone methyl (Fig. 6f).

To correlate NRF2 activity to the progression of HSV-1 infection, we infected HEK 293 cells, transfected with the NRF2 reporter plasmid, with a HSV-1 VP26-mCherry virus and monitored fluorescent protein expression by FACS analysis. This analysis revealed that infected cells with high NRF2 transcriptional activity (population *NRF2* high) displayed lower mCherry signals compared to the *NRF2* low population (Fig. 6g). These results provide further support that high NRF2 activity impairs HSV-1 infection. As an internal control, we defined two populations of cells that were separated perpendicular to the *NRF2* high/low populations (Fig. 6f). The mCherry signal in these two control groups showed no apparent difference (Fig. 6h). In addition, plasmid transfection of HEK 293 cells does not alter VP26-mCherry virus infection efficiency (Supplementary Fig. 6h). Taken together, these experiments confirm the observation from the scRNA-seq data that high NFR2 activity restricts HSV-1 viral infection.

## Discussion

We performed scRNA-seq of primary human fibroblasts at early stages of infection with HSV-1. Using the resolution of scRNA-seq, we showed that cells in the S/G2/M cell cycle phases bear more viral transcripts. The relationship between the cell cycle and the progression of infection has been studied for decades^48–51^, with most reports pointing to a G1 or G1/S arrest upon HSV-1 infection. scRNA-seq now allows for perturbation-free analysis of the relationship between the cell cycle and progression of infection, reducing the risk of experimental artifacts. We have seen that cells in S/G2/M phases on average bear more viral transcripts (Fig. 1f). This is corroborated by recent findings that the activity of *CCNE1* (also known as cyclin E) and *CDK2*, which promote the G1/S transition, correlates with productive infection^52^. Similarly, high levels of the G1/S transition marker *GMNN* (also known as geminin) favored infection in microscopy-based experiments^53^. We observed that, under the conditions used here, the fibroblast populations doubled every 40–50 h. Since about half of the measured cells were in the S/G2/M phases, these would last more than 20 h, much longer than the intervals between infection and harvesting. Our results therefore indicate that cells in S/G2/M phases provide a more favorable cellular environment to establish the infection compared to G1 cells. Extending measurements to later time points could then also detail the cell cycle state in which infected cells are arrested.

Herpes viral genes are classified into immediate early, early, and late^1^. Still, studies so far averaged large numbers of cells, and were not able to distinguish how viral gene expression starts in individual cells. By using clustering of viral gene co-occurence and transition probabilities on diffusion maps, scRNA-seq allowed us to propose a refined model for a set-wise emergence of viral transcripts (Fig. 2), an approach that should be applicable to a number of other viruses. The causes for such a stepwise progression of HSV-1 transcription remain to be investigated. Co-overexpression *UL23* and *UL50* before infection did not alter early viral transcript expression as measured by quantitative reverse transcription PCR (RT-qPCR) (Supplementary Figure 6i). It is therefore likely that the progression of infection is largely shaped by the cellular environment. While our efforts focused on early time points of infection, future studies including later time points may describe previously described modulations of splicing and transcription by the HSV-1 infection^4–6^ into account.

Virus infection leads to changes in host cell transcription. We have identified a number of deregulated genes in bulk RNA-seq; however, only by using the scRNA-seq data we could distinguish how deregulation is related to progression of infection in the same cell. Two small GTPases, *RASD1* and *RRAD*, were directly induced by the infection. We have observed that both possesses antiviral properties (Fig. 3). Interestingly, these two genes are usually not expressed in epithelial cells. They are however putative targets of the primate specific gene *DUX4*^54^. This germline transcription factor was recently shown to be induced in HSV-1-infected cells^55^. Correlating host gene expression with viral gene expression generally emerges as a focus in scRNA-seq studies of virus-infected cells^15,17^, and comparative studies might reveal common topics and modulators of viral infection.

The development of pseudo-time inference algorithms for scRNA-seq data^56^ enabled us to order cells along defined trajectories and made it possible to deduce relationships between biological mechanisms, without artifact-prone perturbances such as gene knockdowns/knockouts. We first used overdispersion analysis^57^ to find genes with inhomogeneous expression across the entire dataset, which might therefore play a role in the infection (Fig. 4). Overdispersion analysis selects genes that have variance of mRNA expression, indicating that these genes are highly expressed in only a subset of cells in the population. The relationship of these genes to the progression of infection was then analyzed using RNA velocity^38^ and graph abstraction. We focused on target genes of the transcription factor *NRF2*, as represented by *NQO1*. Remarkably, in bulk RNA-seq data, these genes do not appear as differentially expressed and, without single-cell data, would therefore likely not be considered for subsequent studies.

*NRF2* has previously been linked to viral infections^58^; however, without a defined function. In a recent influenza virus scRNA-seq study, NRF2 activity was associated with high expression of viral transcripts^15^. For rotavirus, a double-strand RNA virus, *NRF2* activation was recently shown to reduce virus production^59^. Our analysis suggests that NRF2 becomes active in a subset of cells (Fig. 5.) that leads to an escape state, diverging from a progressing lytic infection (Fig. 4). For herpes infections, reports on the role of *NRF2* so far are mixed. Treatment of mice with the *NRF2* agonist *tert*-butylhydroquinone protected them from murine cytomegalovirus infection^60^. In contrast, HSV-1 infection in A549 was drastically reduced upon a 2-day RNA interference of *NRF2*^61^. This treatment, however, induced the antiviral genes, *STING* and *IFI16*, which could confound the direct effect of the *NRF2* depletion. In another study, as observed here for HSV-1, KSHV infection increased NRF2 protein levels^62^.

Following up on these observations, we showed that two *NRF2* agonists, sulforaphane and bardoxolone methyl, the latter currently being in phase 3 clinical trials for treating chronic kidney disease^63,64^, impaired HSV-1 virus replication. Furthermore, a FACS-based reporter assay confirmed that NRF2 activity was correlated with lower efficiency of infection in single cells. At which stage of the infection viral production is impaired, and whether the mere presence of nuclear NRF2, the expression of its target genes, or the interaction of NRF2 with the NF-κB pathway^41^ plays a role remains to be investigated.

In summary, our study provides a detailed analysis of the events at the beginning of HSV-1 infection in primary human fibroblasts. We could relate the activity of single genes to the progression of infection. Using overdispersion and cellular trajectory analysis allowed the identification of relevant pathways, demonstrating how scRNA-seq can provide detailed insight into biological processes influencing viral infections.

## Methods

### Cells and virus

Primary normal human dermal fibroblasts (PromoCell C-12300) were cultured in Dulbecco’s modified Eagle’s medium (DMEM) supplemented with 10% fetal bovine serum, 100 U/ml penicillin, 100 μg/ml streptomycin, and 1% NAEE (ω-oxidize ethyl nonanoate) at 37 °C and 5% CO_2_. Primary cells were cultured until passage 10. Wild-type HSV-1 was derived from a patient isolate and was obtained from Department of Virology, Saarland University Medical School (Homburg, Germany). The same isolate was used in our previous study^5^. The use of primary human HSV-1 isolates was approved by the ethics committee of the “Ärztekammer des Saarlandes” (Saarland Medical Association, approval no. 10/18). Written consent was obtained from the patients prior to the isolation of virus. The virus was propagated in NHDF cells. At 72 hpi, viral supernatant was collected and sterile filtered through a 0.45 μm pore size filter and stored at −80 °C. Viral titers were determined by plaque assay as described previously^5^.

### Infections

Cells were seeded in 10 cm dishes (for scRNA-seq) and in 6-well plates (for bulk RNA samples and slides for immunofluorescence). For the non-synchronized infection at 37 °C, half of the medium (conditioned medium) was removed, and then cells were incubated for 30 min with HSV-1 (MOI 10) or were mock infected. The supernatant was removed and cells were washed with phosphate-buffered saline (PBS), followed by re-applying the conditioned medium. We harvested uninfected cells and at early time points (1, 3, and 5 hpi) in two biological replicates.

Following the same protocol of infection for synchronized infection, cells were incubated for 20 min at 4 °C prior to infection. Virus containing supernatant was also incubated for 1 h at 4 °C. After inoculation cells were washed with PBS and then complete fresh DMEM media was applied and cultured at 37 °C. For Drop-seq all cells were washed with cold PBS and fixed in ice cold methanol (80%).

### Drop-seq scRNA-seq

Cells were fixed and used for Drop-seq single-cell sequencing as previously described^25^. Monodisperse droplets of about 1 nl in size were prepared on a self-built Drop-seq setup following closely the instrument setup and library generation procedure as described^24^. Principle: Upon nanoliter droplet formation, individual cells are co-encapsulated with individual, uniquely barcoded beads, and become lysed. Released polyadenylated RNA molecules then hybridize to polyd(T) primers that are attached to the uniquely barcoded beads. Each captured mRNA molecule is tagged with a barcode, indicating its cell of origin and a UMI. Nanoliter droplets are collected and broken, and RNA molecules are reverse transcribed into complementary DNA (cDNA), amplified by PCR, size selected for mRNA, fragmented, and 3′ ends sequenced in bulk.

cDNA libraries had large average sizes of about 1.5 kb, indicating high-quality RNA and cDNA molecules. Six hundred picograms of each cDNA library was fragmented and amplified for sequencing with the Nextera XT v2 DNA Library Preparation kit (Illumina, San Diego, CA, USA). Single-cell libraries at 1.8 pM (final average insert sizes ranging from 580 to 680 bp) were sequenced in paired-end mode on Illumina Nextseq 500 sequencers using Illumina Nextseq 500/550 High Output v2 kits (75 cycles). Read 1: 20 bp (bases 1–12 cell barcode, bases 13–20 UMI; Drop-seq custom primer 1 Read1CustSeqB), index read: 8 bp, and read 2 (paired end): 64 bp.

### Bulk RNA-seq and analysis

RNA was extracted from cells using Trizol (Thermo Fisher) and the RNA clean and concentrator kit (Zymo). Sequencing libraries were prepared using the TruSeq Stranded mRNA kit (Illumina) according to the manufacturer’s instruction and sequenced on a HiSeq4000 device using 2 × 76 nt paired-end sequencing. Sequencing reads were aligned to the hg38 version of the human genome using tophat2^65^. Standard differential expression analysis was performed using quasR^66^ for counting reads (using the Gencode gene annotation version 28) and edgeR^67^.

### scRNA-seq data processing

The scRNA-seq data was processed using the PiGx pipeline^68^. In short, polyA sequences are removed from reads. The reads are mapped to the hg38 version of the human genome using STAR^69^ with gene models from Gencode version 28. The number of cells, for each sample, is determined using dropbead^25^. Finally, a combined digital expression matrix is constructed, containing all sequenced experiments. This table is available at the GEO entry. Cells containing <2000 detected genes were filtered from the analysis. Raw counts for each cell were normalized by scaling to the total number of UMI, per cell, and log 2 transformed. Normalized distributions were subsequently scaled to have 0 mean, and standard deviation of 1. Principal component analysis was computed based on the scaled expression values of variable genes (defined using FindVariableGenes function). First 20 principal components were used for *T*-distributed stochastic neighbor embedding (tSNE). The analysis was performed using the Seurat package^57^. Cell cycle was assigned to each cell using the CellCycleScoring function. Cell cycle gene sets were taken from ref. ^70^. Normalized total HSV-1 transcription was calculated by summing up raw counts of all detected viral genes, divided by the total number of raw counts, multiplied by a scaling factor of 10,000 and log(2) transformed. Overdispersion analysis was performed using the FindVariableGenes function from the Seurat package. First, the average expression and dispersion are calculated for each gene. Genes are then stratified into 20 categories based on their average expression, and a *z*-score is calculated for dispersion in each of the bins. Genes that have unexpectedly high dispersion for their corresponding average expression value are selected for further downstream analysis. Plots were generated using ggplot2^71^, pheatmap^72^, or ComplexHeatmap^73^.

To demonstrate that the distributional properties of single-cell data did not bias the correlation statistic, we have repeated the correlation analysis in Fig. 3b (*Y*-axis) with variance stabilized data using sctransform^74^. sctransform uses negative binomial regression to remove any apparent relationship between mean and variance, making the data homoscedastic.

Supplementary Figure1d shows correlation of gene expression across cells, with the viral load before (*X*-axis) and after the variance stabilizing transformation (*Y*-axis). Transformed correlation statistic does not differ significantly from the non-transformed statistic, demonstrating that the conclusions based on the correlation statistic were unbiased.

### Diffusion maps

Transition probabilities between cells were estimated based on the diffusion pseudo-time distances^29^, implemented in the destiny Bioconductor package^28^. Diffusion pseudo-time distance between two cells represents the cumulative probability of traveling from one cell to the other on a probabilistic graph. The graph is constructed by embedding cells using gaussian kernels. Obtained distances were converted to transition probabilities using a Laplacian kernel.

### Binning and correlations

Correlations of host cell gene expression with HSV-1 gene expression (Fig. 3), SULF1 or NQO1 expression (Fig. 4), were calculated using binned cells to reduce noise. For this, cells with “high” expression (see Fig. 2b and Supplementary Fig. 4c) were first sorted according to normalized values of HSV-1 transcripts, SULF1 or NQO1. Then, cells were binned with 20 cells per bin. Each bin was then treated as a metacell. Normalized gene expression values for the metacells were calculated by summing up, per gene, all raw values in the bin, dividing by the total raw count in the entire metacell, multiplied by a scaling factor of 10,000 and log(2) transformed. Then, the linear Pearson’s correlation coefficient and slope for each gene or antisense transcript group with HSV-1 transcripts, SULF1 or NQO1, respectively, were calculated.

### RNA interference

NHDF cells were plated in 6-well plates and incubated at 37 °C. After 24 h, cells were transfected with siRNAs (final amount 15 pmol) using Lipofectamine^®^ RNAiMax (Thermo Fisher) according to the manufacturer’s instructions. Briefly, siRNAs were diluted in 125 μL of reduced serum medium (OPTI-MEM I; Invitrogen). The Lipofectamine RNAiMax reagent was diluted in 125 μL of OPTI-MEM I and the two solutions were then mixed and incubated for 10 min at room temperature before addition to the cells. Eight hours after transfection, cells were infected with HSV-1 (MOI 1) as mentioned above. After that cells were again transfected with the siRNAs and RNA samples and cell supernatants were harvested 16 h after infection. siRNAs that were used in this study to transfect NHDF cells are described in Supplementary Table 3.

### Measuring viral DNA in cell culture supernatant

Cell culture supernatant was treated with 2 mg/ml proteinase K (Thermo Fisher) in 30 mM Tris, pH 8, and 0.5% Triton for 10 min at 70 °C, followed by inactivation of the proteinase for 10 min at 95 °C. After 1:1 dilution in water, the samples were used for qPCR using the UL29 primer pair (Supplementary Table 4). A standard curve for relative quantification was prepared from serial dilution of a virus stock with 1 PFU/ml (measured using plaque assays) treated in parallel to the samples. For all experiments, four measurements were done for two biological replicates.

### RT-qPCR

RNA was extracted from cells using Trizol (Thermo Fisher) and the RNA clean and concentrator kit (Zymo). DNase treatment using DNase I amplification grade (Thermo Fisher) and RT using SuperScript III (Thermo Fisher) was performed according to the manufacturer’s protocol. For all experiments, four measurements were done from two biological replicates. Power SYBR Green PCR Master Mix on a StepOnePlus system (both Thermo Fisher Scientific) was used for qPCR. Data were normalized to the indicated timepoint/sample and the GAPDH (glyceraldehyde 3-phosphate dehydrogenase) signal using the ∆∆Ct method^75^. Primers for qPCR were designed using the Universal ProbeLibrary (Roche Life Sciences), tested for efficiency and a single amplicon and listed in Supplementary Table 3.

### Uniform Manifold Approximation and Projection

For visualization purposes, we employed in Figs. 4 and 5 the Uniform Manifold Approximation and Projection (UMAP) dimensionality reduction algorithm^76^ instead of tSNE as in the previous figures. The reason was that the directionality, which the RNA velocity algorithm calculates based on all detectable genes, and then projects as arrow clouds on the two-dimensional map (Fig. 4c), could not be visualized when using tSNE.

Variable genes were defined using the FindVariableGenes function with the default parameters. First 20 principle components were calculated using the defined variable genes. Dimensionality reduction was performed with UMAP^76^. The cells were clustered using Louvain algorithm with a set of resolution parameters ranging from 0.5 to 2.

### Velocity and graph abstraction

The data was pre-processed using the Velocyto CLI pipeline. Only cells with more than 500 intronic and more than 500 exonic reads were kept for further analysis. Intronic signal represented about 3% of all sequencing reads.

The following parameters were used within the Velocyto analysis:

vlm.score_detection_levels()

vlm.filter_genes(by_detection_levels = True)

vlm.score_cv_vs_mean(2000, max_expr_avg = 55)

vlm.filter_genes(by_cv_vs_mean = True)

vlm.normalize_by_total()

vlm.pca = pca(n_components = 10, svd_solver = ‘arpack’, random_state = 1)

vlm.pcs = vlm.pca.fit_transform(vlm.S_norm.T)

vlm.knn_imputation(k = 100, balanced = True, b_sight = 3000, b_maxl = 1500, n_jobs = 16)

vlm.fit_gammas(weighted = False)

vlm.estimate_transition_prob(hidim = “Sx_sz”, embed = “ts”, transform = ‘log’)

vlm.calculate_embedding_shift(sigma_corr = 0.05, expression_scaling = False)

vlm.calculate_grid_arrows(smooth = 0.8, steps = (40, 40), n_neighbors = 100)

For the nascent RNA clustering, the Velocyto estimated transcriptional rates were processed using scanpy^77^. The rates were used to calculate the principal components, UMAP embeddings, cluster the cells using the Louvain algorithm, and compute PAGA^37^.

### Bardoxolone methyl treatment

Bardoxolone methyl was purchased from Sigma-Aldrich (SMB00376) or MedChemExpress (HY-13324). Stock concentrations of 100 and 400 µM were prepared in dimethyl sulfoxide (DMSO), and then diluted 1:1000 into the cell culture medium. Sulforaphane was purchased from Sigma-Aldrich (S4441). Stock concentrations of 2 and 5 mM were prepared in DMSO, and then diluted 1:1000 into the cell culture medium. For the assays combining treatment with HSV-1 infection, half of the medium was removed, virus inoculum at an MOI of 1 was added to the remaining medium, and half an hour later the cells were washed with warm PBS, the conditioned medium added back to the cells with bardoxolone methyl or sulforaphane supplied at the indicated concentrations.

### FACS-based reporter assay

The DNA fragment containing the elements before the *GFP* gene shown in Fig. 6 was synthesized (Biocat) and inserted using *Eco*RI/*Xho*I. The sequence is shown in Supplementary Data 4. The plasmid is available through Addgene. For the assay, HEK 293 cells were transfected within the plasmid using Lipofectamine 2000 (Thermo Fisher), and 24 h later they were infected with HSV-1 *VP26-mCherry*^47^ at an MOI of 1. At 16 hpi, cells were harvested using Trypsin, washed in PBS, filtered through 35 µm cell strainer, and analyzed on a BD Aria Fusion device. Figures were generated using FlowJo version 10. To generate the histograms shown in Fig. 6 and Supplementary Fig. 6a, subsets of equal size were generated using the DownSample v3 plugin.

### Cloning of *UL23*/*UL50*/*UL33*/*US2* expression constructs

Coding sequences of these HSV-1 genes were synthesized as codon-optimized variants (Biocat). The sequences are listed in Supplementary Data 4. To generate expression constructs with either an N-terminal or C-terminal StrepHA tag, the sequences were cloned into previously described vectors^78^ using *Kpn*I/*Not*I restriction enzymes. The plasmids are available through Addgene. Note that qPCR primer pairs used to detect viral UL23 and UL50 do not detect the codon-optimized overexpression constructs.

### Reporting summary

Further information on research design is available in the Nature Research Reporting Summary linked to this article.

## Supplementary information

Supplementary Information

Peer Review File

Reporting Summary

Description of Additional Supplementary Files

Supplementary Data 1

Supplementary Data 2

Supplementary Data 3

Supplementary Data 4

Source Data File

## Data Availability

Raw sequencing reads as well as raw read counts for both the bulk RNA-seq and the scRNA-seq, along with normalized counts and tSNE coordinates and cell cycle information, are available in the NCBI GEO repository, accession number GSE123782. Data underlying Figs. 3a–c, 6a–c, Supplementary Figs. 6a–e, i are provided in the Source Data file, and, for Fig. 3a, in Supplementary Data 3. Viral read counts are provided in Supplementary Data 2. All other data are available from the corresponding author upon requests.

## Code availability

Custom R, Python, Perl, awk, and bash scripts are readily available from the authors upon request.
