# Supplementary material for: Single-cell RNA-sequencing of herpes simplex virus 1-infected cells connects NRF2 activation to an antiviral program
Source: Nat Commun. 2019 Oct 25;10:4878. doi: 10.1038/s41467-019-12894-z (PMC6814756; doi:10.1038/s41467-019-12894-z)
Supplement: Supplementary file 1 — Supplementary Information [file 41467_2019_12894_MOESM1_ESM.pdf]

Supplementary Information

Single-cell RNA-sequencing of Herpes simplex virus 1-infected cells connects NRF2 activation to an antiviral program

Wyler et al.

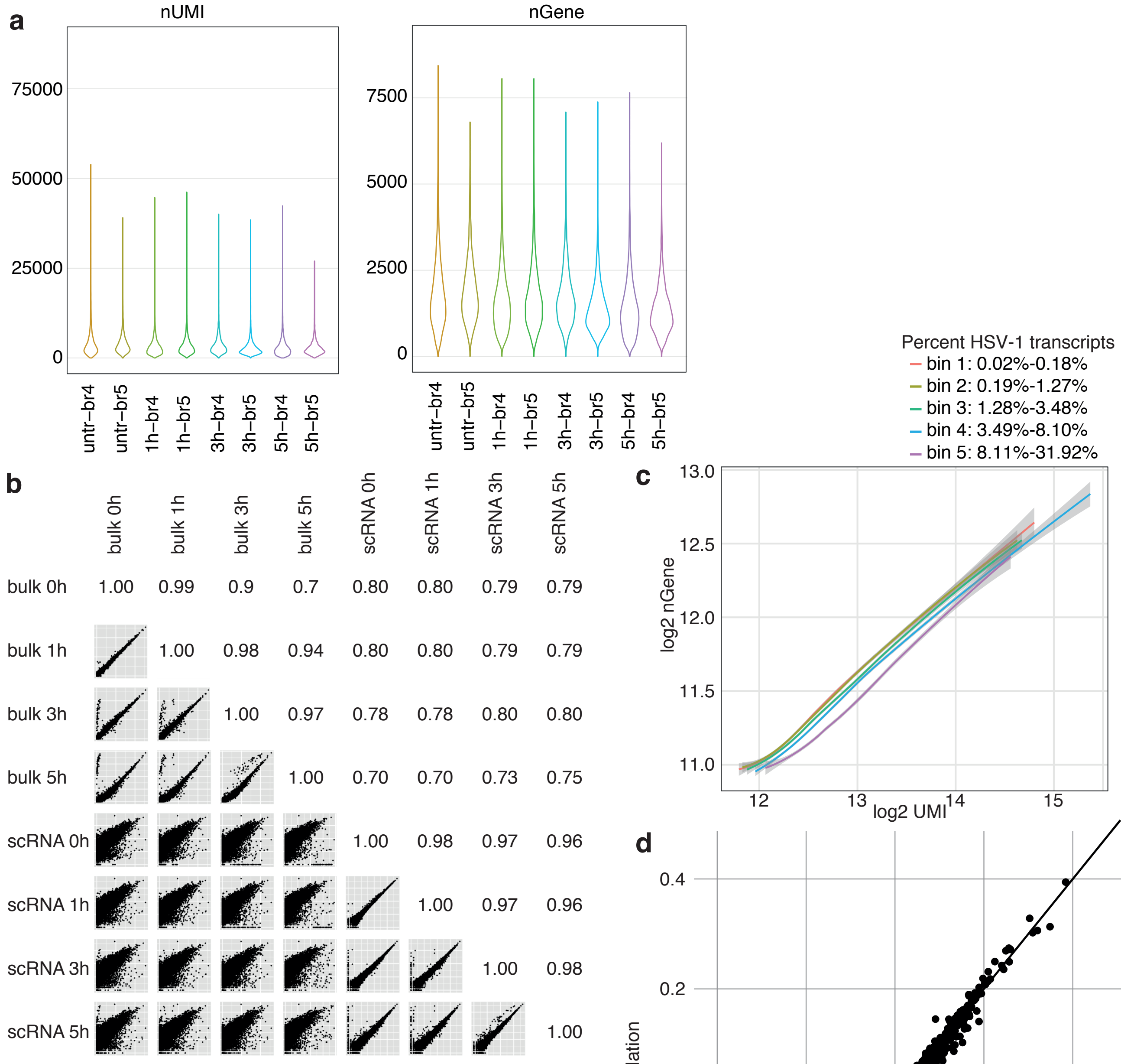

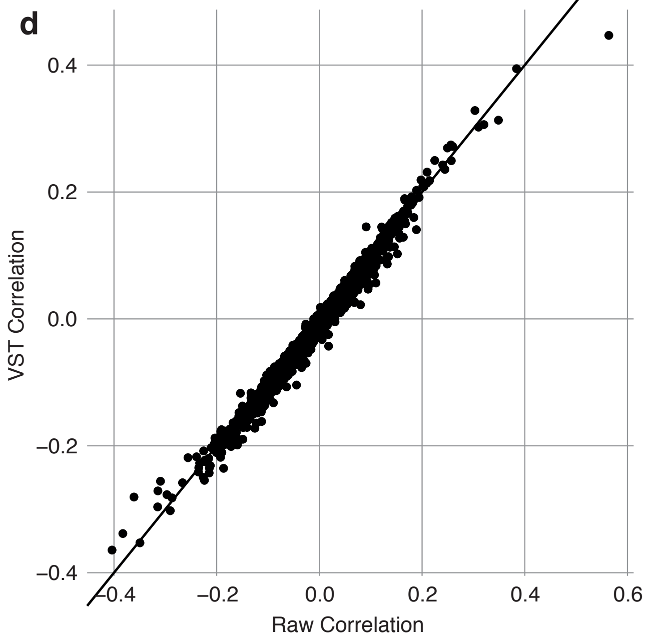

Supplementary Figure 1 Statistical summary of scRNA-seq data
a, Violin plots showing the distribution of number of unique molecular identifiers and number of detected genes in the individual scRNA-seq samples. **b**, Correlation of bulk RNA-seq with scRNA-seq data. Shown are scatter plots (lower left part) and linear correlation coefficients (upper right part) of bulk polyA RNA-seq values (horizontal axes, log(2) transformed fpkm values) and scRNA-seq values (vertical axes, log(2) transformed sums of scRNA-seq counts) of merged replicates, **c**, Cells harvested at 5hpi containing at least one viral transcript were binned according to the percentage of viral transcripts per cell, and the log2 transformed nUMI/n-Gene values plotted as smoothed means with 95% confidence intervals in gray. Percentage ranges of the bins are indicated below the plot. **d**, Correlation of host cell genes with viral transcripts using raw data (horizontal axis) and variant-stabilized (VST) correlation (vertical axis).

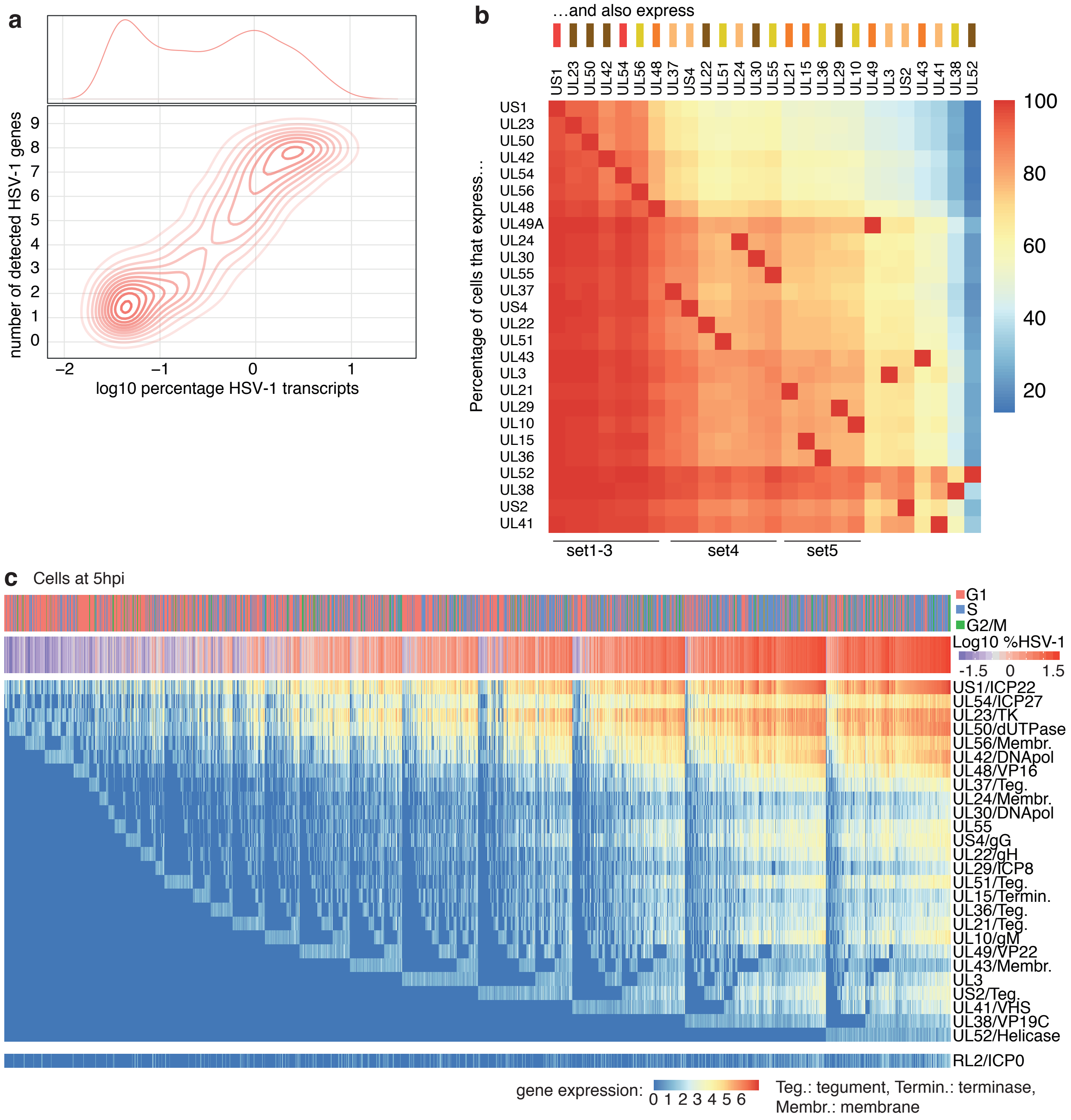

Supplementary Figure 2 Viral expression at the 5hpi time point and diffusion maps **a**, Smoothened two-dimensional densities of the number of detected viral genes (vertical axis) in relationship to the percentage of viral transcripts (horizontal axis) for the set of cells used for Fig. 2b. Relative densities as in Fig. 1e are shown on top. **b**, Percentages indicating co-occurences of genes as in Fig. 2c. **c**, Heatmap of expression values of the first eight expressed viral genes for experiment two with only "HSV-1 high" 5hpi cells as defined in Fig. 2b. Rows (genes) and columns (cells) were sorted as described in the main text for Figure 3b. Above the heatmap, cell cycle and log10 transformed percentage of viral transcripts are shown. **d**, Clustered heatmap of transition probabilities on the diffusion maps of "HSV-1 high" cells harvested at 1hpi and 3hpi. Left part, clustered probabilities colored from white (low) to red (high). Columns and rows represent cells in the same order. Right part, per cluster the percentage of cells with non-zero values for the respective gene/gene transcription is shown as a heatmap. The dotted rounded rectangle denote clusters of transition probabilities between the states as detailed in the bottom part. For this analysis, only S/G2/M phase cells were used, since the difference between G1 and non-G1 single-cell transcriptomes otherwise dominates the transition probabilites. **e**, As in d but with cells harvested at 3hpi and 5hpi.

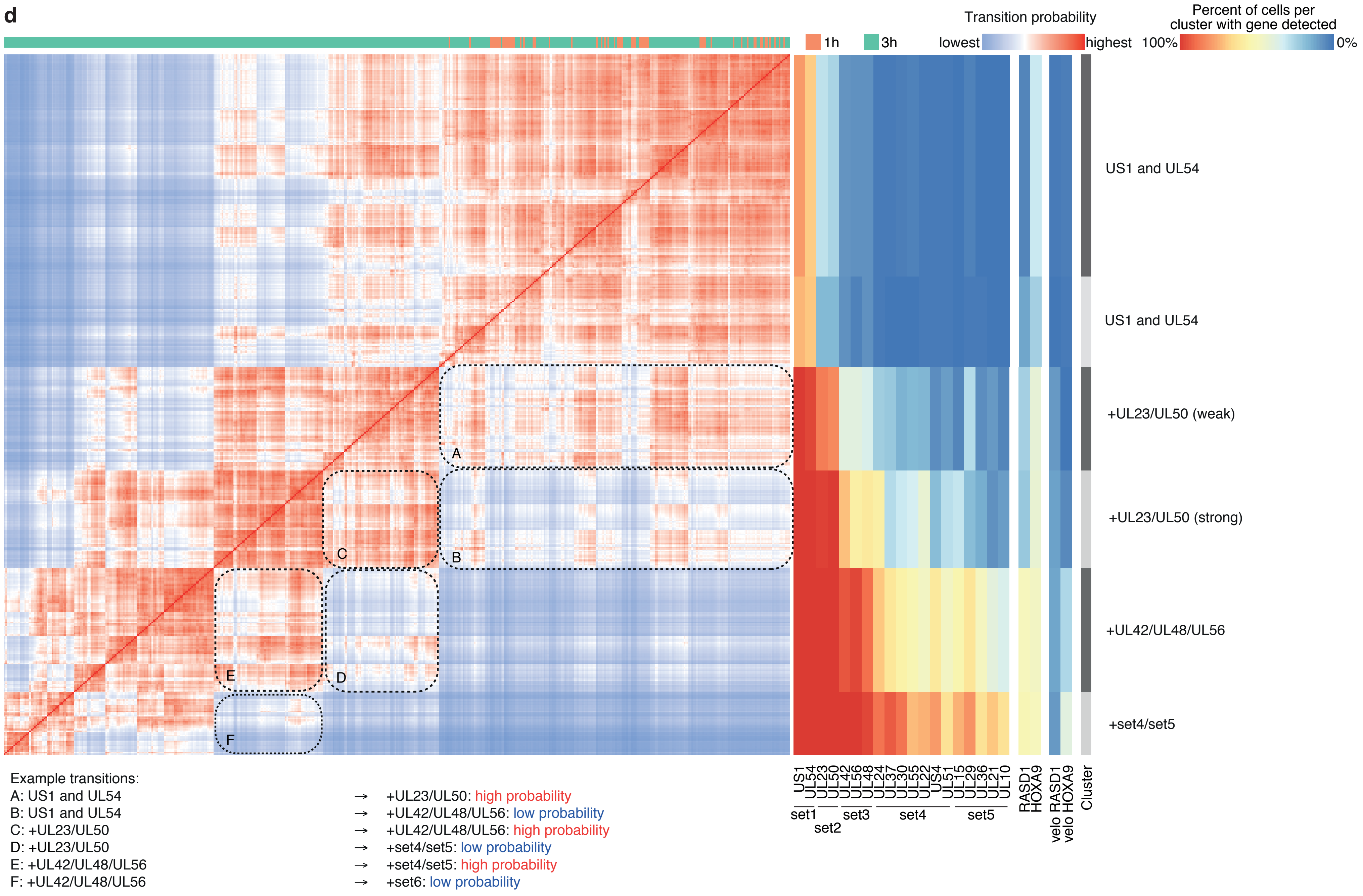
d
1h
3h
Transition probability
lowest
highest
Percent of cells per cluster with gene detected
100%
0%
A
B
C
D
E
F
US1 and UL54
US1 and UL54
+UL23/UL50 (weak)
+UL23/UL50 (strong)
+UL42/UL48/UL56
+set4/set5
US1
UL54
UL23
UL50
UL42
UL56
UL48
UL24
UL37
UL30
UL55
UL22
US4
UL51
UL15
UL29
UL36
UL21
UL10
RASD1
HOXA9
velo RASD1
velo HOXA9
Cluster
set1
set2
set3
set4
set5
Example transitions:
A: US1 and UL54 → +UL23/UL50: high probability
B: US1 and UL54 → +UL42/UL48/UL56: low probability
C: +UL23/UL50 → +UL42/UL48/UL56: high probability
D: +UL23/UL50 → +set4/set5: low probability
E: +UL42/UL48/UL56 → +set4/set5: high probability
F: +UL42/UL48/UL56 → +set6: low probability

e

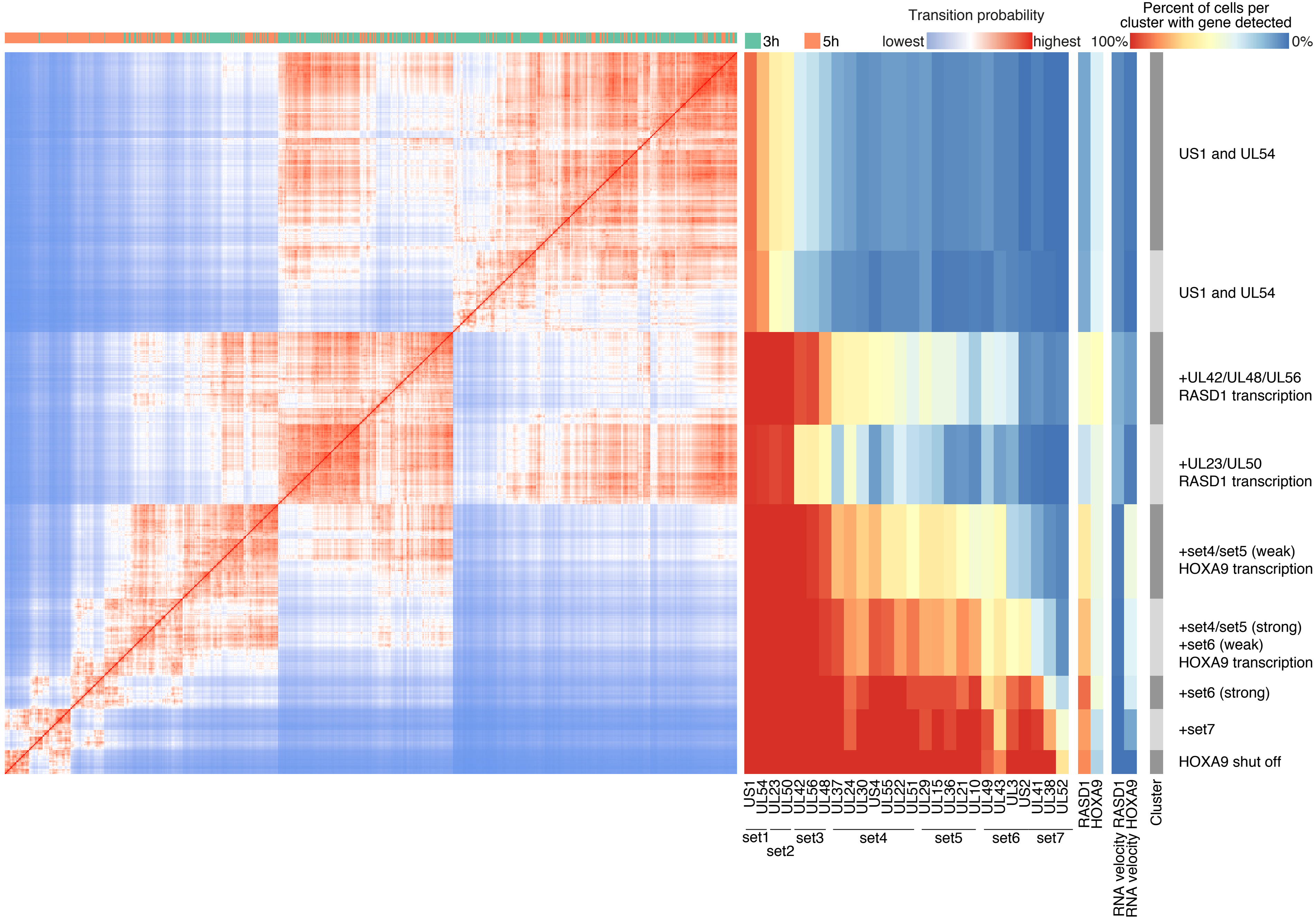

3h
5h
Transition probability
lowest
highest
Percent of cells per cluster with gene detected
100%
0%
US1 and UL54
US1 and UL54
+UL42/UL48/UL56
RASD1 transcription
+UL23/UL50
RASD1 transcription
+set4/set5 (weak)
HOXA9 transcription
+set4/set5 (strong)
+set6 (weak)
HOXA9 transcription
+set6 (strong)
+set7
HOXA9 shut off
US1 UL54 UL23 UL50 UL42 UL56 UL48 UL37 UL24 UL30 US4 UL55 UL22 UL51 UL29 UL15 UL36 UL21 UL10 UL49 UL43 UL3 US2 UL41 UL38 UL52
set1
set2
set3
set4
set5
set6
set7
RASD1
HOXA9
RNA velocity RASD1
RNA velocity HOXA9
Cluster

a

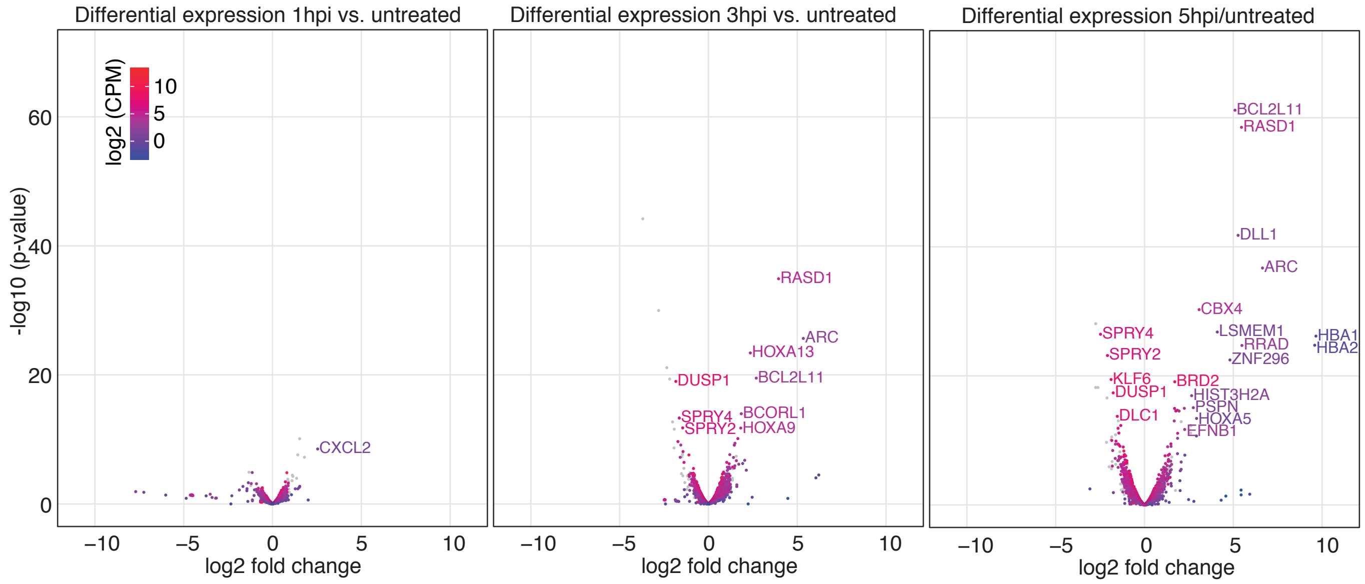

Supplementary Figure 3 Differential gene expression in bulk RNA-sequencing **a**, Differential bulk mRNA expression comparing expression from 1hpi (left panel), 3hpi (middle panel), and 5hpi (right panel) with untreated cells. Outliers were marked by name. Horizontal axis denote log(2) transformed fold-changes, vertical axis log(10) transformed p-values. The color represents the log(2) transformed counts per millions over all time points. Low-reproducibility genes (see Supplementary Table S3) are displayed as light gray dots.

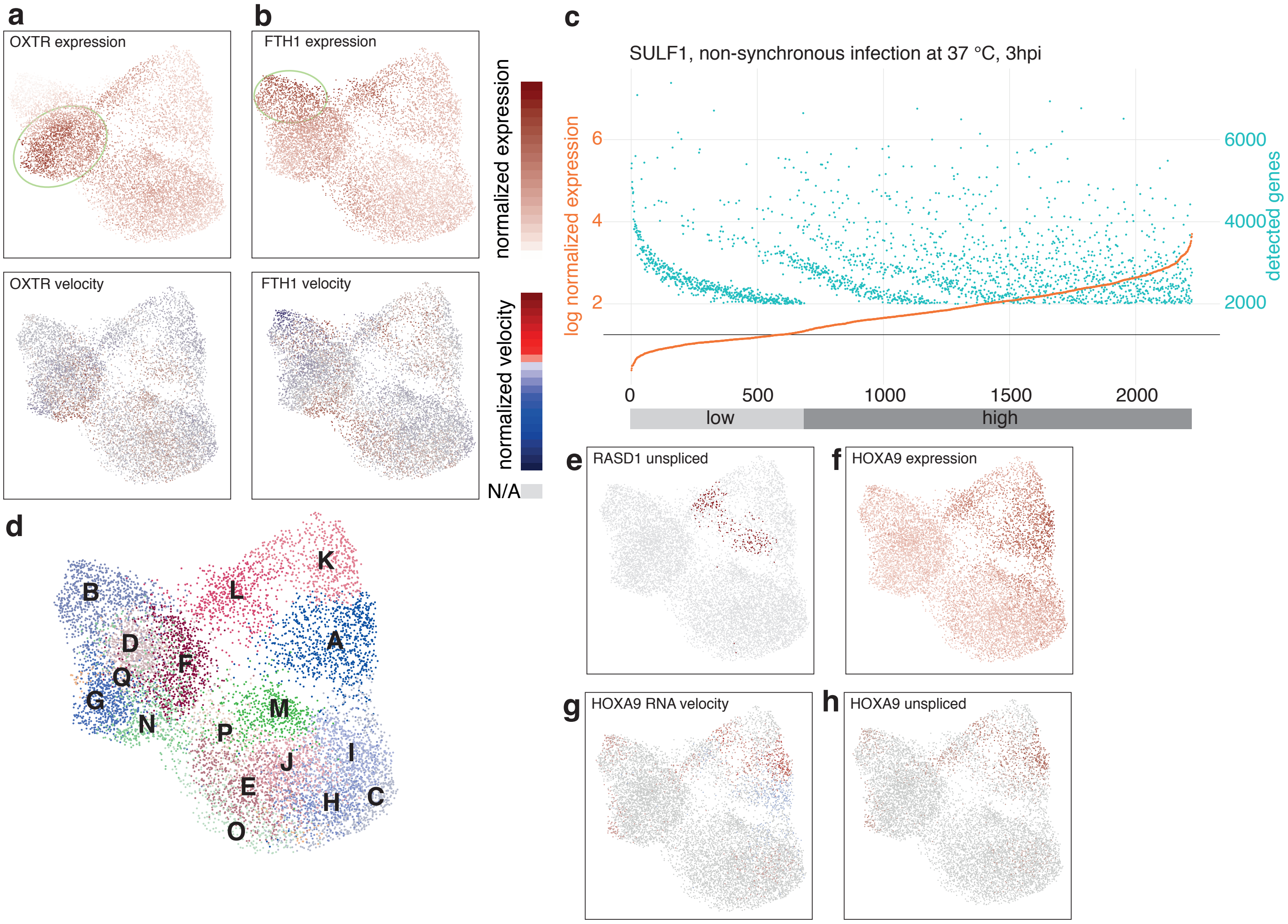

Supplementary Figure 4 Transcriptional bursts in infected cells **ab**, Cells colored by expression values (top panels) and RNA velocity values (bottom panels) of the indicated genes. Color scales are shown to the right of these panels. For expression values, areas containing cells with relatively high expression levels were marked with a light green ellipse. For RNA velocity, cells for which no value could be calculated were colored in light gray. **c**, Cells harvested at 3hpi were sorted by normalized SULF1 transcript UMIs. For each cell, the red dot represents the log(2) transformed amount of SULF1 transcript UMIs (left axis), and the green dot the number of genes (human and viral) detected (right axis). Note that only cells with more than 2000 detected genes were used, thus the lower apparent limit for the green dots. Light and dark gray bars designate low SULF1 cells and high SULF1 cells according to the bimodality of the plot. The horizontal gray line denotes the cutoff expression between high and low. Only high SULF1 cells were used to calculate the correlations in Fig. 4h. **d**, Cells colored by PAGA clusters from Fig. 4i. **e-h**, Cells collored by the presence of unspliced RASD1 (e), mature HOXA9 (f), HOXA9 RNA velocity (g) and unspliced HOXA9 (h).

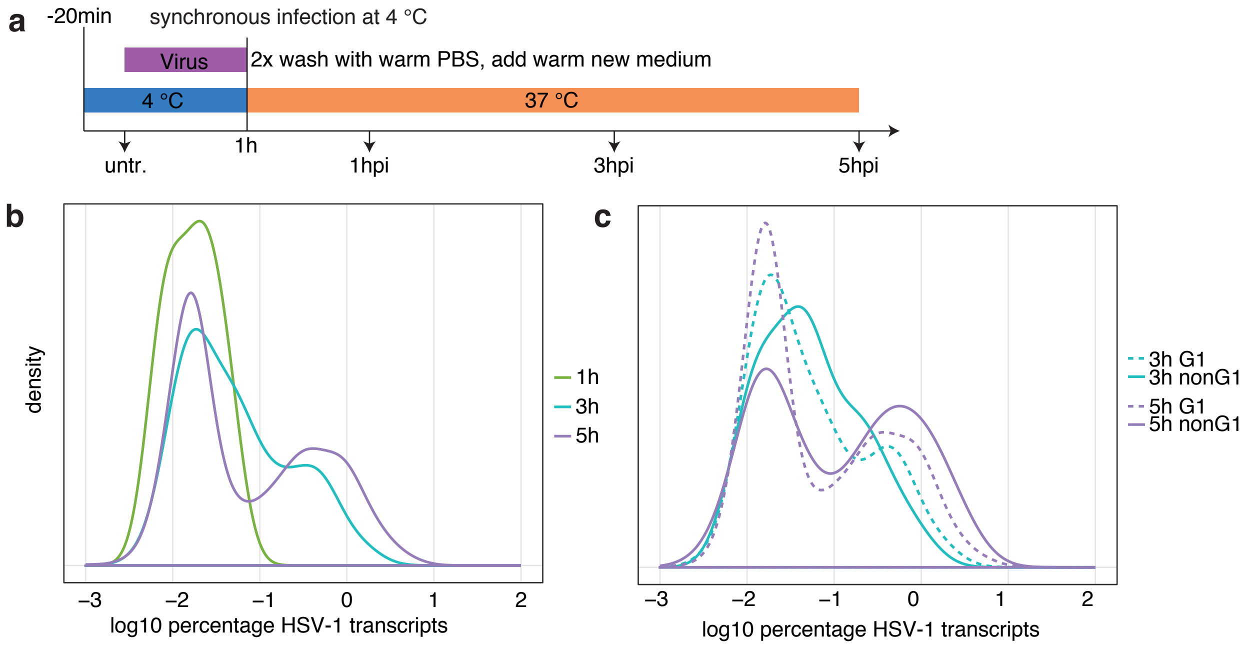

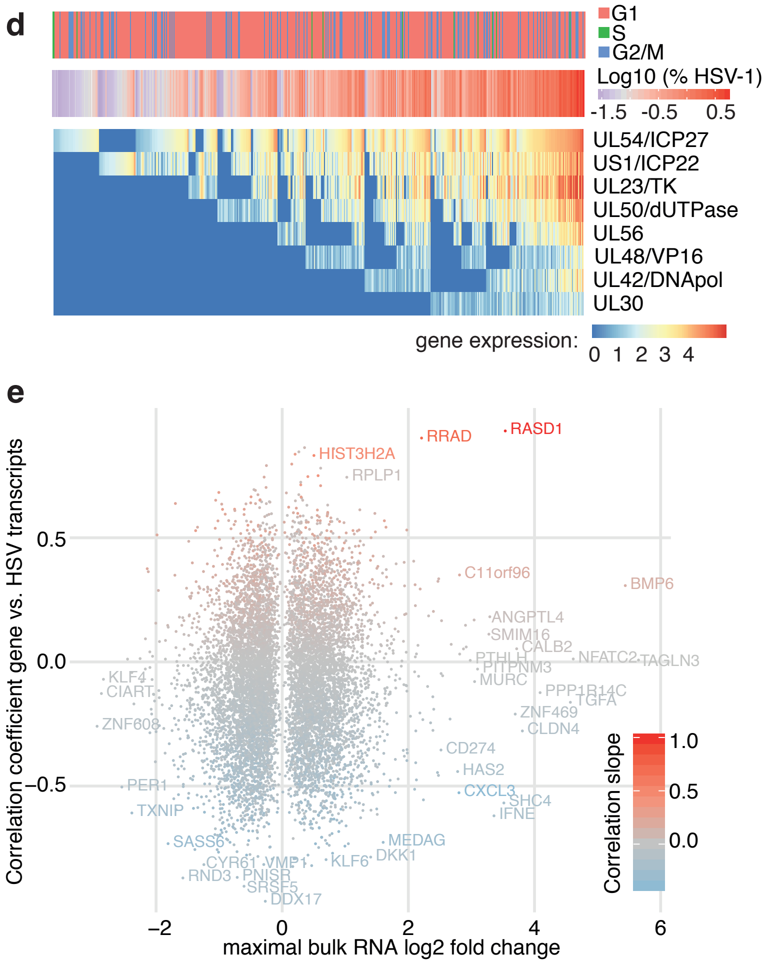

Supplementary Figure 5 Independent experiment using a synchronized infection protocol **a**, Infection protocol for the synchronized infection at 4 °C. In parallel to single-cell RNA-sequencing, cells were harvested for bulk mRNA-sequencing and ICP0 immunofluorescence staining. **b**, Relative densities of the percentage of viral transcripts (log10 transformed) per cell for the three time points post infection. **c**, Relative densities of the percentage of viral transcripts per cell (log10 transformed) for G1 and non-G1 cells for cells harvested at 3hpi and 5hpi. **d**, Heatmap of expression values of the first eight expressed viral genes for experiment two with only "HSV-1 high" cells. Rows (genes) and columns (cells) were sorted as described in the main text for Figure 2. Above the heatmap, cell cycle and log10 transformed percentage of viral transcripts are shown. **e**, Relationship between differentially expressed genes and viral transcription. The horizontal axis shows the maximal log(2) transformed bulk RNA-seq fold change of the three time points after infection compared to uninfected cells, the vertical axis the linear correlation coefficient of the gene expression with the sum of viral transcripts in bins of 20 cells, using only "HSV-1 high" cells. **f**, Cells were projected on a two-dimensional map using UMAP according to gene expression values, with cells colored by harvesting time point. Main distinguishing features were marked in grey. **g**, Coloring by amount of HSV-1 transcripts. Cells without HSV-1 transcripts are colored in light gray. **h**, cells colored by expression values (top panels) and RNA velocity values (bottom panels) of the indicated genes. Color scales are shown to the right of these panels. For expression values, areas containing cells with relatively high expression levels were marked with a light green ellipse. The dark green and blue ellipse denote S and G2/M phase cells, respectively. For RNA velocity, cells for which no value could be calculated were colored in light gray. **i**, Correlation of gene expression with NQO1 expression (horizontal axis) and SULF1 expression (vertical axis) in cells harvested at 3hpi. Every dot represents a gene, colored by the slope of the linear correlation with NQO1. Selected genes were labeled by name. Note that gene names were slightly jittered to improve readability. **j**, Groups of cells according to PAGA were labeled A to S. Transition probabilities between clusters are proportional to the line thickness between the clusters. Areas of interest are marked with light green ellipses.

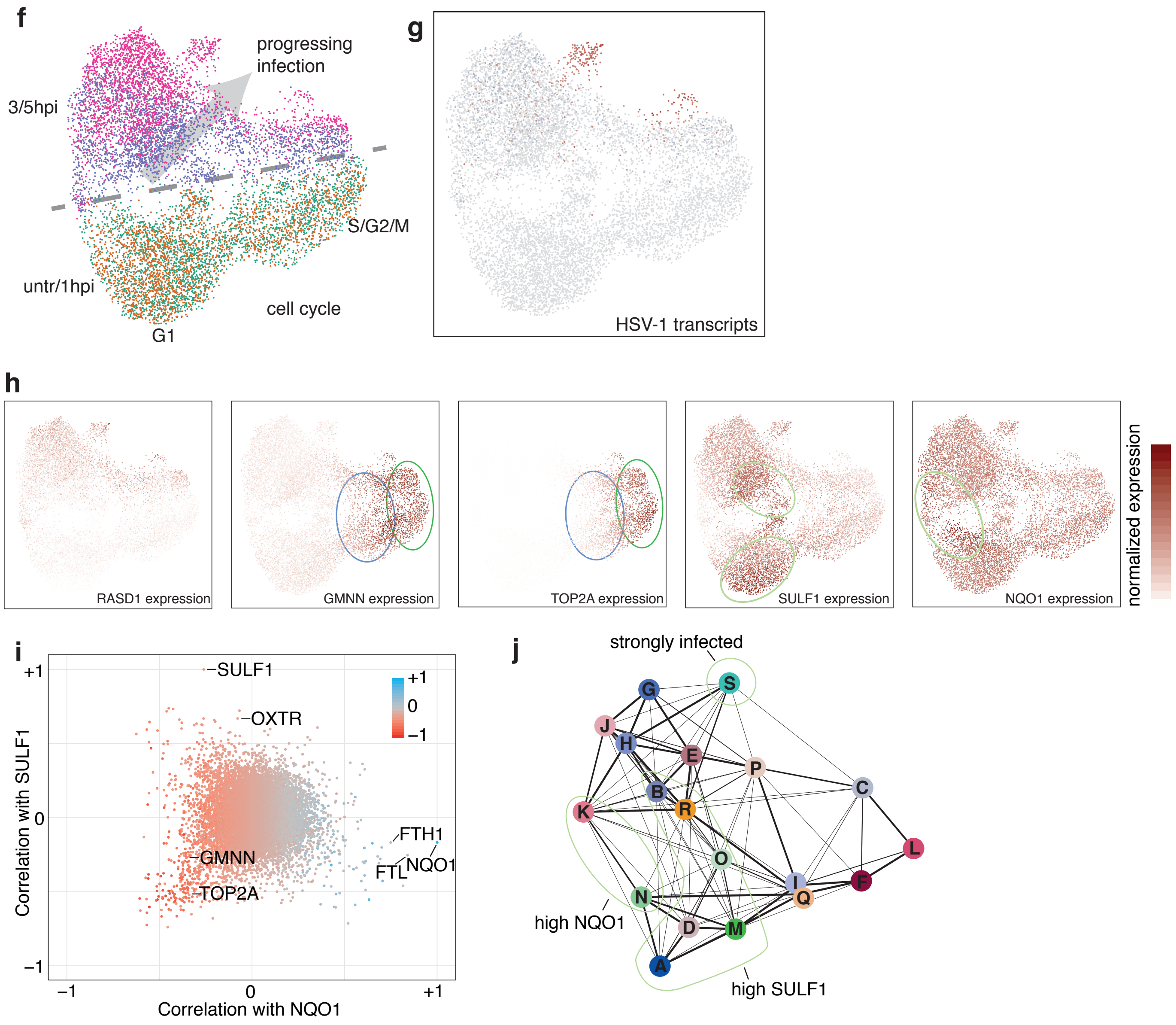
f
progressing infection
3/5hpi
S/G2/M
untr/1hpi
cell cycle
G1
g
HSV-1 transcripts
h
RASD1 expression
GMNN expression
TOP2A expression
SULF1 expression
NQO1 expression
normalized expression
i
SULF1
OXTR
FTH1
FTL
NQO1
GMNN
TOP2A
Correlation with SULF1
Correlation with NQO1
+1
0
−1
j
strongly infected
high NQO1
high SULF1
S
G
J
H
E
P
C
B
K
R
L
O
I
F
N
Q
D
M
A

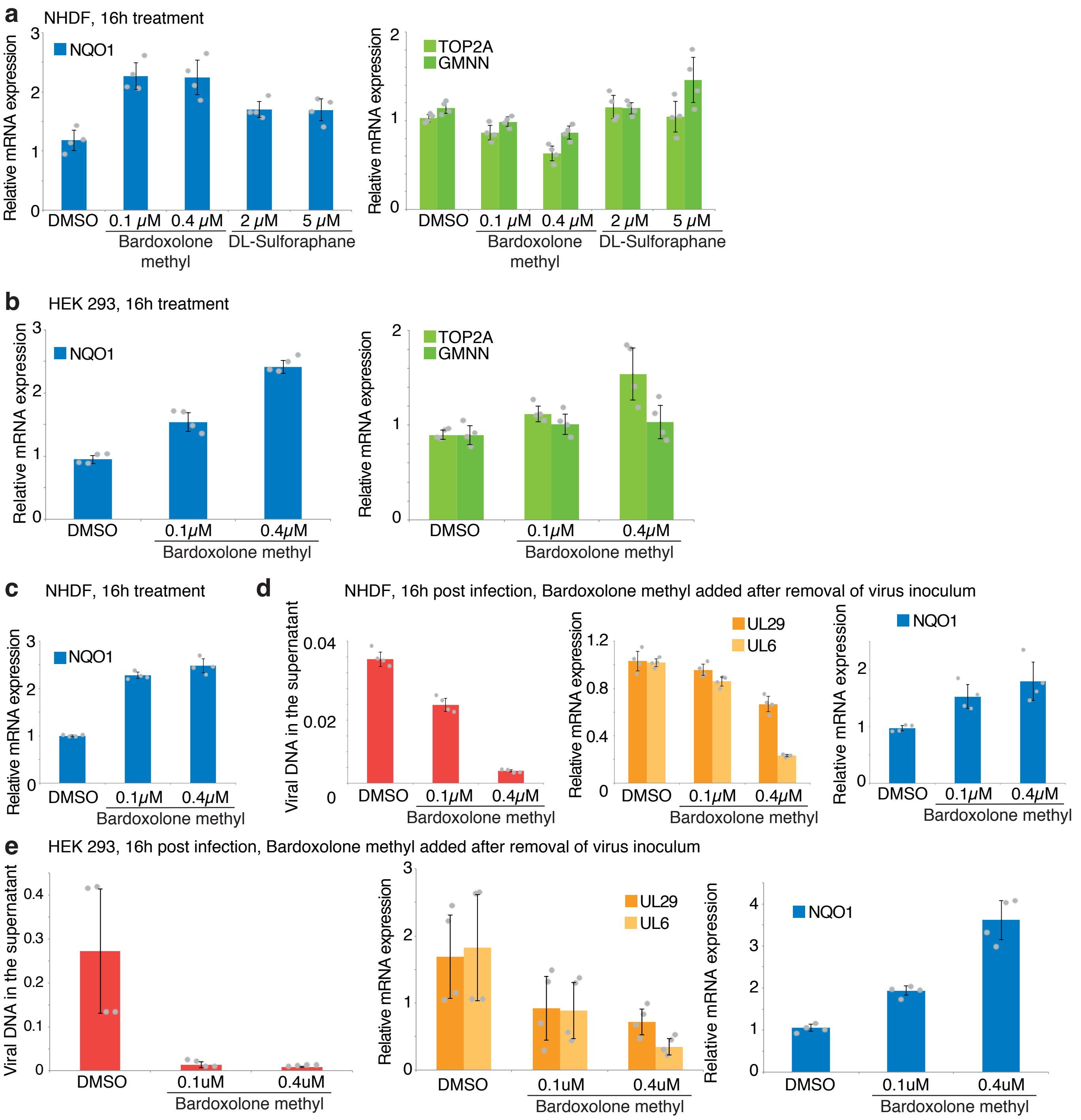

Supplementary Figure 6 NRF2 agonists induce NQO1 and are antiviral also in HEK 293 cells; FACS gating strategy; UL23/UL50 overexpression. **ab**, NHDF(a) and HEK 293 (b) cells were treated for 16h with solvent (DMSO) or different concentrations of Bardoxolone methyl/Sulforaphane, followed by RNA isolation. NQO1, TOP2A and GMNN mRNA levels were probed using RT-qPCR. **cd**, As in Figure 6a-c, but with a different source of Bardoxolone methyl (MedChemExpress solution in DMSO instead of powder from Sigma-Aldrich). **e**, HEK 293 cells were infected with HSV-1 at an MOI of 1. After removal of virus inoculum and washing with PBS, conditioned medium supplied with solvent or different concentrations of Bardoxolone methyl wasadded. At 16hpi, viral DNA was probed in the supernatant using qPCR (left panel) and viral mRNAs and NQO1 in the RNA isolated from the cells using RT-qPCR (right panel). For all panels, barplots indicate means, error bars denote standard deviations, the individual measurement values are shown as grey dots. **f**, gating strategy to separate populations of cells by FACS. Viable, single cells were first selected based on FSC/SSC values (not shown). Cells with BFP signals above a certain threshold were defined as transfected. Cells with mCherry signals above a certain threshold were defined as mCherry+. Transfected cells were then either separated in the “NRF2 low” (low GFP to BFP ratio) and “NRF2 high” (high GFP to BFP ratio) populations, or control populations. The control populations were defined by separating populations perpendicular to the separation between “NRF2 low” and “NRF2 high”. The following populations were used to create the histograms in Fig. 6 and this Supplementary Figure. Fig. 6e and Supplementary Fig. 6g, GFP and BFP signal and the ratio GFP/BFP in transfected cells, without any infection. Fig. 6f, mCherry signal in all viable, single cells from infected, but not transfected cells. Fig. 6g, mCherry signal from the NRF2 low and the NRF2 high subpopulations according to the scheme in Supplementary Fig. 6f. Fig. 6h, mCherry signal from the control 1 and the control 2 subpopulations according to the scheme in Supplementary Fig. 6f. **g**, histogram of GFP (left) and BFP (right) values from which the ratios in Fig. 6e were calculated. **h**, HEK 293 cells were either transfected with the reporter plasmid (light green) or left untreated before infection. Shown is the distribution of mCherry values at 16hpi as determined by FACS. **i**, HEK 293 cells were transfected with expression constructs for UL33 and US2 (control), or all possible combinations of N-terminally and C-terminally tagged UL23 and UL50, and 24h later infected with an MOI of 3. Shown are viral transcript levels assessed at 3hpi using RT-qPCR (left) and a Western blot showing expression of all tagged viral protein (right).

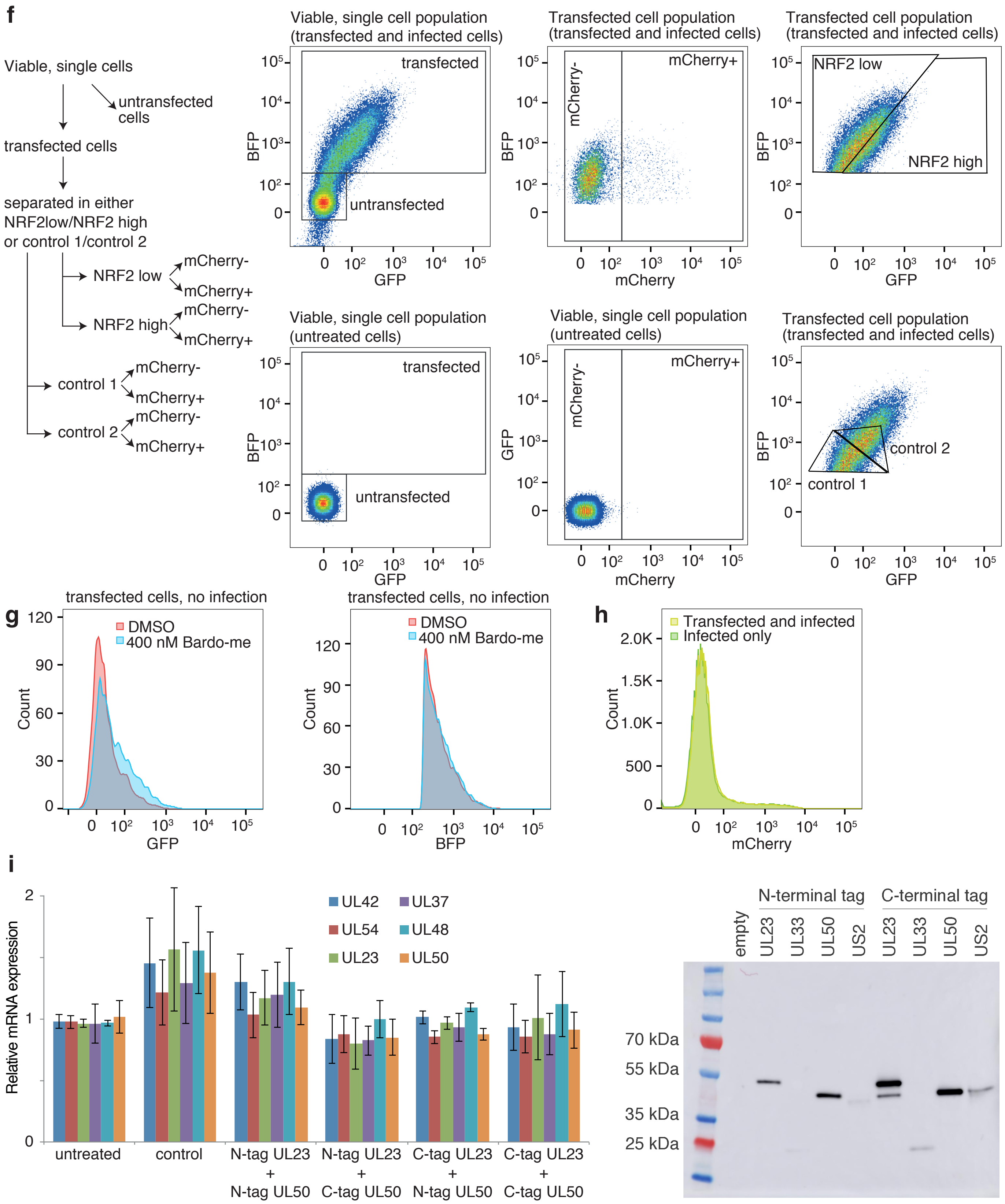
f
Viable, single cells
untransfected cells
transfected cells
separated in either NRF2low/NRF2 high or control 1/control 2
NRF2 low
NRF2 high
control 1
control 2
mCherry-
mCherry+
Viable, single cell population (transfected and infected cells)
transfected
untransfected
Transfected cell population (transfected and infected cells)
NRF2 low
NRF2 high
Viable, single cell population (untreated cells)
control 1
control 2
BFP
GFP
mCherry
g
transfected cells, no infection
DMSO
400 nM Bardo-me
Count
h
Transfected and infected
Infected only
i
Relative mRNA expression
UL42
UL37
UL54
UL48
UL23
UL50
untreated
control
N-tag UL23 + N-tag UL50
N-tag UL23 + C-tag UL50
C-tag UL23 + N-tag UL50
C-tag UL23 + C-tag UL50
N-terminal tag
C-terminal tag
empty
UL23
UL33
UL50
US2
70 kDa
55 kDa
35 kDa
25 kDa

Supplementary Table 1: Samples

Pilot experiment (not analyzed in manuscript): Replicate 1, synchronized 4 °C infection

Replicate 1 - 0h (untreated cells)	scRNA-seq Rep 1 0hpi		
Replicate 1 - 3hpi	scRNA-seq Rep 1 3hpi		
Replicate 1 - 5hpi	scRNA-seq Rep 1 5hpi		

Replicate 2 and 3, synchronized 4 °C infection

Replicate 2 - 0h (untreated cells)	scRNA-seq Rep 2 0h	bulk polyA RNA-seq Rep 2 0h	ICP0 Immunofluorescence (not shown)
Replicate 2 - 1hpi	scRNA-seq Rep 2 1h	bulk polyA RNA-seq Rep 2 1h	ICP0 Immunofluorescence (not shown)
Replicate 2 - 3hpi	scRNA-seq Rep 2 3h	bulk polyA RNA-seq Rep 2 3h	ICP0 Immunofluorescence (not shown)
Replicate 2 - 5hpi	scRNA-seq Rep 2 5h	bulk polyA RNA-seq Rep 2 5h	ICP0 Immunofluorescence (not shown)
Replicate 3 - 0h (untreated cells)	scRNA-seq Rep 3 0h	bulk polyA RNA-seq Rep 3 0h	ICP0 Immunofluorescence (not shown)
Replicate 3 - 1hpi	scRNA-seq Rep 3 1h	bulk polyA RNA-seq Rep 3 1h	ICP0 Immunofluorescence (not shown)
Replicate 3 - 3hpi	scRNA-seq Rep 3 3h	bulk polyA RNA-seq Rep 3 3h	ICP0 Immunofluorescence (not shown)
Replicate 3 - 5hpi	scRNA-seq Rep 3 5h	bulk polyA RNA-seq Rep 3 5h	ICP0 Immunofluorescence (not shown)

Replicate 4 and 5, non-synchronized 37 °C infection, conditioned medium

Replicate 4 - 0h (untreated cells)	scRNA-seq Rep 4 0h	bulk polyA RNA-seq Rep 4 0h	ICP0 Immunofluorescence (not shown)
Replicate 4 - 1h	scRNA-seq Rep 4 1h	bulk polyA RNA-seq Rep 4 1h	ICP0 Immunofluorescence (not shown)
Replicate 4 - 3h	scRNA-seq Rep 4 3h	bulk polyA RNA-seq Rep 4 3h	ICP0 Immunofluorescence (not shown)
Replicate 4 - 5h	scRNA-seq Rep 4 5h	bulk polyA RNA-seq Rep 4 5h	ICP0 Immunofluorescence (Fig. 1B)
Replicate 5 - 0h (untreated cells)	scRNA-seq Rep 5 0h	bulk polyA RNA-seq Rep 5 0h	ICP0 Immunofluorescence (not shown)
Replicate 5 - 1h	scRNA-seq Rep 5 1h	bulk polyA RNA-seq Rep 5 1h	ICP0 Immunofluorescence (not shown)
Replicate 5 - 3h	scRNA-seq Rep 5 3h	bulk polyA RNA-seq Rep 5 3h	ICP0 Immunofluorescence (not shown)
Replicate 5 - 5h	scRNA-seq Rep 5 5h	bulk polyA RNA-seq Rep 5 5h	ICP0 Immunofluorescence (not shown)
Mock - 0h (untreated cells)		bulk polyA RNA-seq Mock 0h	
Mock - 1h		bulk polyA RNA-seq Mock 1h	
Mock - 3h		bulk polyA RNA-seq Mock 3h	
Mock - 5h		bulk polyA RNA-seq Mock 5h	

Supplementary Table 2: cells with more than 500/2000 detected genes

Pilot experiment: Replicate 1, synchronized 4 °C infection, more than 500 genes detected

	0 hpi	1 hpi	3 hpi	5 hpi	Sum per row
Replicate 1	3713	N/A	3634	8370	15717

quantified cells with more than 2000 genes detected

	0 hpi	1 hpi	3 hpi	5 hpi	Sum per row
Replicate 1	888	N/A	1308	1508	3704
With HSV-1 genes	0	N/A	72	482	
Percent with HSV-1 genes	0	0	5,50%	31,96%	

Replicate 2 and 3, synchronized 4 °C infection, more than 500 genes detected

	0 hpi	1 hpi	3 hpi	5 hpi	Sum per row
Replicate 2	4409	5106	4500	4850	18865
Replicate 3	5802	6681	5640	5766	23889
Sum per column	13924	11787	13774	18986	58471
With HSV-1 genes	0	0	440	4423	

quantified cells with more than 2000 genes detected

	0 hpi	1 hpi	3 hpi	5 hpi	Sum per row
Replicate 2	1158	1300	1520	1405	5234
Replicate 3	2131	1642	1795	1495	6960
Sum per column	3289	2942	3315	2900	15764
With HSV-1 genes	0	5	134	870	
Percent with HSV-1 genes	0	0,17%	4,04%	30,00%	

Replicate 4 and 5, non-synchronized 37 °C infection, conditioned medium, more than 500 genes detected

	0 hpi	1 hpi	3 hpi	5 hpi	Sum per row
Replicate 4	4409	5106	4500	4850	18865
Replicate 5	5802	6681	5640	5766	23889
Sum per column	13924	11787	13774	18986	58471
With HSV-1 genes	0	0	440	4423	

quantified cells with more than 2000 genes detected

	0 hpi	1 hpi	3 hpi	5 hpi	Sum per row
Replicate 4	1813	1858	1601	1030	6302
Replicate 5	1993	1887	1166	973	6019
Sum per column	3806	3745	2767	2003	12321
With HSV-1 genes	0	450	2238	2001	
Percent with HSV-1 genes	0	12,02%	80,88%	99,90%	

Supplementary Table 3: Oligonucleotides

siRNAs

siRASD1-3	CCGCGAUGAUCAAGAAGAUdTdT
siRASD1-4	CGACACAACCUAAGGAGGAdTdT
siRRAD-2	CCAUUGUAGUGGACGGAGAdTdT
siRRAD-3	AGGCAUCACUCAUGGUCUAdTdT

qPCR primers

Name	Sequence	Amplicon length
CXCL8_fwd	agacagcagagcacacaagc	62 bp
CXCL8_rev	atggttccttccggtggt	
GAPDH_fwd	agccacatcgctcagacac	66 bp
GAPDH_rev	gcccaatacgaccaaatcc	
GMNN_fwd	gcatctggatctcttgttgga	126 bp
GMNN_rev	ttcactagattctgggacaataacc	
IL11_fwd	agctgcaaggtcaagatggt	61 bp
IL11_rev	agattgtttccagtttgctatgg	
NQO1_fwd	cggctttgaagaagaaaggat	110 bp
NQO1_rev	cgcagggtccttcagtttac	
RASD1_fwd	cctctccatcctcacaggag	114 bp
RASD1_rev	aggcaagacttggtgtcgag	
RRAD_fwd	gacgagagcgtttacaaggtg	129 bp
RRAD_rev	ggagcgatcataggtgtgc	
TOP2A_fwd	tccacgatacatctttacaatgc	126 bp
TOP2A_rev	aaaaacttcaacgtgtgatcatct	
UL29_fwd	ccatcatctcctcgcttagg	63 bp
UL29_rev	agctgcagatcgaggactg	
UL6_fwd	aggacggctgggtaaaggt	60 bp
UL6_rev	ggagaatctcgcggaacag	
UL42_fwd	ggcatccttatccataacacg	68 bp
UL42_rev	ctgaattgcgagtgttccag	
UL54_fwd	agacgcctcgtccgacgga	74 bp
UL54_rev	gaggcgcgaccacacactgt	
UL23_fwd	gcgggtttatatagacggtcct	104 bp
UL23_rev	tcggctcgggtacgtaga	
UL37_fwd	gggagacgtcgctcaagat	85 bp
UL37_rev	gggaagagcaggctgtacg	
UL48_fwd	cccgacctgtttgactgc	77 bp
UL48_rev	aacatgaagggctggaacag	
UL50_fwd	gctgacttttgcgcgattat	79 bp
UL50_rev	ccgagtcgataagacccagt	
