# Supplementary material for: Single-cell RNA-sequencing of herpes simplex virus 1-infected cells connects NRF2 activation to an antiviral program
Source: Nat Commun. 2019 Oct 25;10:4878. doi: 10.1038/s41467-019-12894-z (PMC6814756; doi:10.1038/s41467-019-12894-z)
Supplement: Supplementary file 4 — Description of Additional Supplementary Files [file 41467_2019_12894_MOESM4_ESM.pdf]

Description of Additional Supplementary Files

File Name: Supplementary Data 1
Description: Highly variable genes omitted and/or flagged in downstream analyses.

File Name: Supplementary Data 2
Description: Total nUMIs, raw viral read count, normalized viral read count for cells with more than 2000 detected genes.

File Name: Supplementary Data 3
Description: Correlation of host genes with viral transcription

File Name: Supplementary Data 4
Description: Sequence of the reporter plasmid insert, codon-optimized sequences used for the UL23/UL50/UL33/US2 expression plasmids.
