# Supplementary material for: Single-cell RNA-sequencing of herpes simplex virus 1-infected cells connects NRF2 activation to an antiviral program
Source: Nat Commun. 2019 Oct 25;10:4878. doi: 10.1038/s41467-019-12894-z (PMC6814756; doi:10.1038/s41467-019-12894-z)
Supplement: Supplementary file 2 — Peer Review File [file 41467_2019_12894_MOESM2_ESM.pdf]

Reviewers' comments:

Reviewer #1 (Remarks to the Author):

In the manuscript by Wyler et al., the authors profiled the transcriptomes at a single-cell level of HSV1-infected primary human fibroblasts and non-infected controls. The analysis identified a range of genes that were differentially regulated. Using different time-points after infection, the authors produce a temporal overview of the order of which genes are regulated by the infection. They use this analysis to determine that cells clustered based on time post infection, cell-cycle markers, and viral mRNA content. The authors then move on to identify RRAD and RASD1 as differentially regulated genes – as has also been demonstrated on several previous accounts (also stated by the authors). A single approach based on siRNA is then used to support that these genes contribute to resistance to the virus – measured only on viral DNA content. The role of these cell-cycle genes are then not addressed further. The authors then move on to identify SULF1 and NQO1 as being negatively correlated – suggesting that Nrf2 (which drives NQO1 transcription) might be important for HSV resistance. Unfortunately, the authors do not address this claim thoroughly. Again, only a single experiment using an NRF2 inducer is used. The conclusion is therefore weak.

In conclusion, the connection between cell-cycle-state and resistance to HSV1 infection is indeed interesting. The manuscript, however, is mostly based on correlations and speculations by single-cell transcriptome analysis and therefore mostly descriptive in nature. The few smaller attempts to move beyond correlation by siRNA experiments are extremely preliminary and not well controlled. Thus, I cannot recommend publication of this manuscript in Nature Communications

Reviewer #2 (Remarks to the Author):

In this ms Wyler et al analyzed in single cell the initial stages of HSV infection. The amount of data that has been collected and the thorough analysis are very impressive. There are many potentially interesting observations but since the audience that will be most interested in these findings are herpesvirus virologists, the authors should make more effort to present their data in a way that will be more intuitive and accessible to an average virologist. Below are several suggestions.

The authors discuss the relationship between high levels of HSV-1 mRNA and lower nUMI count and that this relates to host cell shut-off. Presumingly they refer to human genes UMIs but this needs to be clarified in the text and in the figures (is detected genes in figure S2 and S4 refers only to host genes?)

The authors state the “viral gene transcription appears to start mostly around the internal repeats, and around gene UL23 (Fig. 2a).” However no transcripts expression is presented from the repeat regions. Is this an alignment issue? In addition, expression of ICP4 and ICP0, two central immediate early genes are not captured as in the first eight expressed viral genes. Can the authors comment on that?

It seems like the authors also detect late gene expression. Can they use similar analysis as the one presented in Figure S2 to identify barriers for late gene expression?

Figure S2d (part 2) that contains the analysis from which Figure 2C was derived, should be better explained.

Do the single cell analysis supports the previous definition of IE/E and L genes or are there genes that seems to have different kinetics then was previously suggested?

The authors indicate that “cells with high NQO1 levels, and therefore high preceding NRF2 activity, had a relatively low probability to progress into the infection, compared to cells from groups D and F”. What are groups D and F? Please clarify. Also do cells that express high levels of SULF1 that anti correlate with NQO1 have high probability to progress into infection?

It seems from the velocity analysis that RASD1 transcription is being shut off in the most infected cells (although total expression is high). Is that the case? If yes, the authors should discuss how this reflects on RASD1 function

The authors should include few words that will clarify what are overdispersion and velocity analyses.

The valocity analysis is based on the identification of intron reads. HSV was reported to disrupt host gene splicing and was recently also shown to induce read-through. Do the authors identify disruptions in splicing/transcription termination in their scRNA-deq data? could they use their data to correlate these phenomena with specific viral transcripts? how these effects (if indeed detected) will effect the velocity analysis?

Personally I find the viral bottlenecks the authors have identified as the most interesting finding. The authors should at least discuss the functions of UL23 and UL50 and how these potentially act as bottlenecks. Maybe an interesting experiment will be to over express these proteins and to test if this affects infection compared to over-expressing of viral genes that the authors predict they do not serve as as major bottlenecks.

In general it will be of great value if the authors can generate accessible tables that will contain some of the data about viral and cellular gene expression from their single cells (so it could be used as a fruitful ground for future research). For example a table that contain all the viral reads from the single cells. A table that summaries the differential expression of the bulk RNA—seq analysis. A table with the correlation co-efficient of viral genes with host genes etc…

Reviewer #3 (Remarks to the Author):

Summary:
Wyler and co-workers used single-cell RNA sequencing of fibroblasts infected with HSV-1 over several timepoints in order to identify cellular processes take place during early-infection stages. They applied dimensionality reduction techniques on the sequencing data to gain insights associated with early viral infection. They identified cell cycle dependency, viral gene expression cascades, anti-viral genes, and importantly NRF2 activation as anti-viral pathways. They went on to show that NRF2 inducer, Bardoxolone methyl can impair HSV-1 infection. The methodology and finding are interesting. However, certain parts of the manuscript and figures are poorly presented. Additional validations and method clarification could further strengthen the manuscript.

1. The authors should validate the finding that NRF2 activation as an antiviral mechanism in a subpopulation of cells using cells harboring an NRF2 reporter. Infect the cells with HSV-1, perform flow cytometry live cell sorting to sort out NRF2 activated cell and corresponding NRF2 inactive cells. Then, the authors should be able to follow and compare those cell populations to see if the NRF2 activated population are indeed less infected with virus.
2. In addition to Bardoxolone methyl, the authors should use additional NRF2 inducers coupled with the use of NRF2 knockout cells to cross validate their findings. These experiments are important to make sure that the observed antivirus effect is not specific to bardoxolone methyl and is NRF2 dependent.

3. Please include a data transformation section in the materials and methods section. Please demonstrate homoscedasticity of the transformed data used to calculate linear regression correlation coefficient.
4. Figure 2a is not clear. Please describe the meaning of the horizontal dotted line. Is this a threshold by which subsequent genes were selected?
5. Figure 2b: Please explain why was UL24 discounted, considering it passed the dotted line threshold.
6. Figure S2A should be moved to main Figure 2 to make it easier for readers to understand the definition of “HSV-1 high”.
7. Figure 2b and Figure S2b are confusing. Is Figure S2B necessary?
8. Line 142, shouldn’t that be referring to Fig. S2A?
9. Figure 3d: y-axis title is misleading. For example “0.08 viral DNA in supernatant” does not make much sense. Please consider revising that y-axis title to PFU/ml.
10. Watch spelling: e.g., line 33 “transcrips” = “transcripts”
11. Line 96: what is nUMI defined as? Acronyms should be spelled out in the main text.
12. Figure 2: “HSV-1 high” definition should be supplied in main text and/or figure legend, and not in supplemental. Figure needs to be able to stand on its own without referencing supplemental information to interpret the figure.
13. Line 126-127: recommend changing “start mostly around” to “start in regions flanking the internal repeat regions”.
14. Figure 2a: Please label the bars in the IRL and IRS regions with their gene(s)
15. Figure 2b, legend: “experiment two” is an unclear definition. Also, “HSV-1 high” is not defined in main text results section, and the figure should stand alone without referring to supplemental figures. Supp Fig 2 seems particularly important and perhaps should be moved to main text.
1. Temper the wording in lines 139-141: while I agree with the authors’ conclusions that generally UL1 and UL54 are transcribed prior to UL34 and UL50, there are notable subpopulations that expression UL34 and/or UL50 in the absence of UL1 and UL54. So it is not universal that US1 and UL54 were transcribed before 23 and 50 (line 140).
2. Line 142: “as shown above” in the main text should be “as shown in Fig. 2a”?
3. Line 142: I do not see a bimodal distribution in Fig. 2a, and Fig. 2a; is this actually referring to Fig 1e?
4. Lines 683-684: refers to Figures 3 and 4, not 4 and 5?
5. Gene names should be italicized

## Reply to reviewers

We would like to thank the reviewers for their constructive criticism and insightful suggestions. Additionally, we greatly appreciate the positive and supportive comments.
One reviewer said “the connection between cell-cycle-state and resistance to HSV1 infection is indeed interesting.”
Another reviewer stated that “the amount of data that has been collected and the thorough analysis are very impressive.”
The third reviewer mentioned “the methodology and finding are interesting, however, certain parts of the manuscript and figures are poorly presented.”

A common criticism was that there are many potentially interesting observations but since the audience that will be most interested in these findings are herpesvirus virologists, the authors should make more effort to present their data in a way that will be more intuitive and accessible to an average virologist.
Furthermore, one reviewer mentioned that additional validations and method clarification could further strengthen the manuscript.

The major changes to the manuscript:

- We have revised and extended the investigation of the temporal ordering of viral gene expression. We could bring together the two applied data analysis methods, namely the occurrence of viral genes and the diffusion maps. As a conclusion, we proposed a refined model of viral transcription based on the set-wise emergence of specified viral genes.
- We have substantially extended the follow-up experiments on the influence of NRF2 activity on HSV-1 infection by adding another NRF2 agonist and a FACS-based reporter assay as proposed by Reviewer #3. We would like to emphasize that a key feature of our study is how the influence of NRF2 on HSV-1 infection is derived from the single-cell data using state-of-the-art analytical tools and then followed-up and confirmed by a series of experiments.
- On several occasions, we have increased the detail of the explanations. We also would like to make the data easily accessible for other researchers and provide several data tables accessible on the NCBI GEO database and/or as supplemental tables.

We now present a revised manuscript that has addressed each of the issues raised by the reviewers. Major changes within the manuscript are marked in yellow.

### Point-by-point response to Reviewers’ comments:

### Reviewer #1:

**... In conclusion, the connection between cell-cycle-state and resistance to HSV1 infection is indeed interesting. The manuscript, however, is mostly**

**based on correlations and speculations by single-cell transcriptome analysis and therefore mostly descriptive in nature. The few smaller attempts to move beyond correlation by siRNA experiments are extremely preliminary and not well controlled.**
The goal of our effort was to provide a detailed picture of temporal events and cellular heterogeneity in HSV-1 infected cells. To this end, we generated a comparatively very deep single cell expression data set, consisting of tens of thousands of cells, in multiple biological replicates. On the other hand, we agree that for a data-heavy method such as single-cell RNA-sequencing, the output can be largely descriptive. However, we think that the main strengths of the manuscript, beyond providing a large resource for HSV-1 researchers, are:
1) Our approach of deriving the temporal order of viral gene expression by using temporal scRNA-seq in infected cells and diffusion map analysis will be of great interest to the virology community for studying gene expression cascades of other viruses (especially in genetically non-tractable viruses). We revised the entire section of the viral gene expression cascade and propose a refined model of set-wise expression of viral gene expression that could only be inferred from scRNA-seq data.
2) The observation of the cellular heterogeneity of responses in virus-infected cells and the identification relevant biological processes from deep single-cell RNA-seq data, as demonstrated for the antiviral role of NRF2 in HSV-1 infection. We now strengthened these findings by adding a second NRF2 agonist and a FACS-based readout for NRF2 activity and infection efficiency (see detailed response to reviewer 3 (3a) further below).

## Reviewer #2:

**2a. The authors discuss the relationship between high levels of HSV-1 mRNA and lower nUMI count and that this relates to host cell shut-off. Presumingly they refer to human genes UMIs but this needs to be clarified in the text and in the figures (is detected genes in figure S2 and S4 refers only to host genes?)**
In the result section (second last paragraph of the first section), we refer to both host cell and viral genes, and have clarified this. The effect would be stronger with only human genes, but we feel that representing only human genes could be misleading. Also in Supplementary Fig. 2 (now Fig. 2) and Supplementary Fig. 4, these are both host cell and viral genes, which was clarified in the Figure legends.

**2b. The authors state the “viral gene transcription appears to start mostly around the internal repeats, and around gene UL23 (Fig. 2a).” However, no transcripts expression is presented from the repeat regions. Is this an alignment issue?**
As suggested by reviewer #3, we have clarified this sentence to “start in regions flanking the internal repeat regions”.

**2c. In addition, expression of ICP4 and ICP0, two central immediate early genes are not captured as in the first eight expressed viral genes. Can the authors comment on that?**

We first ensured that ICP4 (RS1) and ICP0 (RL2) are not underrepresented in the scRNA-seq data compared to the bulk RNA-seq. In an earlier paper (Harkness et al. 2014), the authors found also very low sequencing read counts for ICP4 and ICP0. We think that due to the unusually high GC content, the two respective mRNAs are less efficiently detected compared to other viral RNAs (in scRNA-seq as well as bulk sequencing), which makes comparisons difficult. We added two sentences on this observation in the first result section, and included RL2/ICP0 expression in Fig. 2d.

**2d. It seems like the authors also detect late gene expression. Can they use similar analysis as the one presented in Figure S2 to identify barriers for late gene expression?**
As suggested, we extended the diffusion map analysis to late infection stages. We indeed found these barriers, i.e. a step-wise progression of viral gene expression. See also response to the reviewer comment 2k below, were we describe in detail the changes made to Fig. 2.

**2e. Figure S2d (part 2) that contains the analysis from which Figure 2C was derived, should be better explained.**
The entire section has been rewritten (see also response to reviewer comment 2k below), containing more verbose explanations.

**2f. Does the single cell analysis support the previous definition of IE/E and L genes or are there genes that seems to have different kinetics then was previously suggested?**
The analysis overall corroborates the previous classification of genes into the IE/E and L classes. However, the previous classification can now be further refined based on the single cell measurements. In particular, we think that this approach of deriving the temporal order of viral gene expression by using temporal scRNA-seq in infected cells and diffusion map analysis will be of great interest to the virology community to study gene expression cascades of other viruses (especially in genetically non-tractable viruses).

**2g. The authors indicate that "cells with high NQO1 levels, and therefore high preceding NRF2 activity, had a relatively low probability to progress into the infection, compared to cells from groups D and F". What are groups D and F? Please clarify. Also do cells that express high levels of SULF1 that anti correlate with NQO1 have high probability to progress into infection?**
The letters refer to the groups in Fig. 4i. The group with high NQO1 levels is group B. Group D is the one with high levels of SULF1. This description has been clarified in the text. Furthermore, we added relative probability values in Fig. 4i, and coloring of cells by PAGA groups in Fig. S4d.
We have also re-evaluated our observations and conclusions regarding SULF1. Since we now feel that our interpretations presented in the first version of the manuscript are too speculative, we deleted parts of the RNA velocity section of the revised manuscript.

**2h. It seems from the velocity analysis that RASD1 transcription is being shut off in the most infected cells (although total expression is high). Is that**

**the case? If yes, the authors should discuss how this reflects on RASD1 function.**

This is an important suggestion and was very helpful for a deeper analysis of host cell transcription during infection. We added supplemental data on unspliced reads from RASD1 and the newly added gene HOXA9 in Supplementary Fig. 4, and we have discussed the findings in the discussion. Basically, the data show that there are sequential transcription bursts for RASD1 and HOXA9 during the development of infection. This can also be seen in the newly designed Supplementary Fig. 2e. In there, it can be seen how these transcriptional bursts correspond to specific stages of the infection, and coincide with the emergence of specific viral genes. Also, as in Supplementary Fig. 4, it becomes evident that these bursts are sequential in time.

On one side, the shutoff of RASD1 transcription is unlikely to be caused by the general shutoff of transcription, since HOXA9 is transcribed in cells that do not transcribe RASD1 anymore. However, the last cluster in Supplementary Fig. 2e, where the transcription of both RASD1 and HOXA9 cannot be detected, represents a state in which host cell transcription is largely shut off.

On the other side, since two temporally distinct groups of cells transcribe RASD1 or HOXA9, different mechanisms likely contribute to the transcriptional inductions of these two genes.

Furthermore, the transcriptional burst for RASD1 is sufficient to create enough mRNA so that it is detectable through the later stages of infection. With the RASD1 protein having likely a longer half-life than the RASD1 mRNA, the activity of RASD1 can therefore reach far longer than the transcriptional burst. Studying this would require a sensitive single-cell proteomics approach, which is not yet feasible. Beyond the transcription itself, we therefore hesitate to draw any time-related conclusions on RASD1 activity.

**2i. The authors should include few words that will clarify what are overdispersion and velocity analyses.**

We have added additional explanation at the beginnings of the respective result sections.

**2j. The velocity analysis is based on the identification of intron reads. HSV was reported to disrupt host gene splicing and was recently also shown to induce read-through. Do the authors identify disruptions in splicing/transcription termination in their scRNA-deq data? could they use their data to correlate these phenomena with specific viral transcripts? how these effects (if indeed detected) will effect the velocity analysis?**

We greatly appreciate this insightful comment. Indeed, the perturbation of splicing and/or transcription by HSV-1 infection could impact the RNA velocity analysis. Under the conditions analyzed here, we observed only a slight increase of intronic reads at 5hpi compared to uninfected cells in the RNA-sequencing data (see Fig. R1 below), whereas this becomes very well detectable at later time points, e.g. the ones measured in Rutkowski et al. 2015. Furthermore, as detailed above in reviewer comment 2h, we exemplified two genes, RASD1 and HOXA9, with transcriptional bursts. If there would be a broad increase in the number unspliced reads, we would not observe bursts but a gradual increase in the transcriptional activity. On the other side, we could indeed correlate these

transcriptional bursts with the emergence of specific viral transcripts (Supplementary Fig. 2e).
Therefore we assume that at the time points chosen for our study, disruptions in RNA synthesis and processing are not yet pronounced enough to become detectable in the single-cell data. Nevertheless, we have added this issue to the discussion, since it should be considered for further studies employing also later time points.

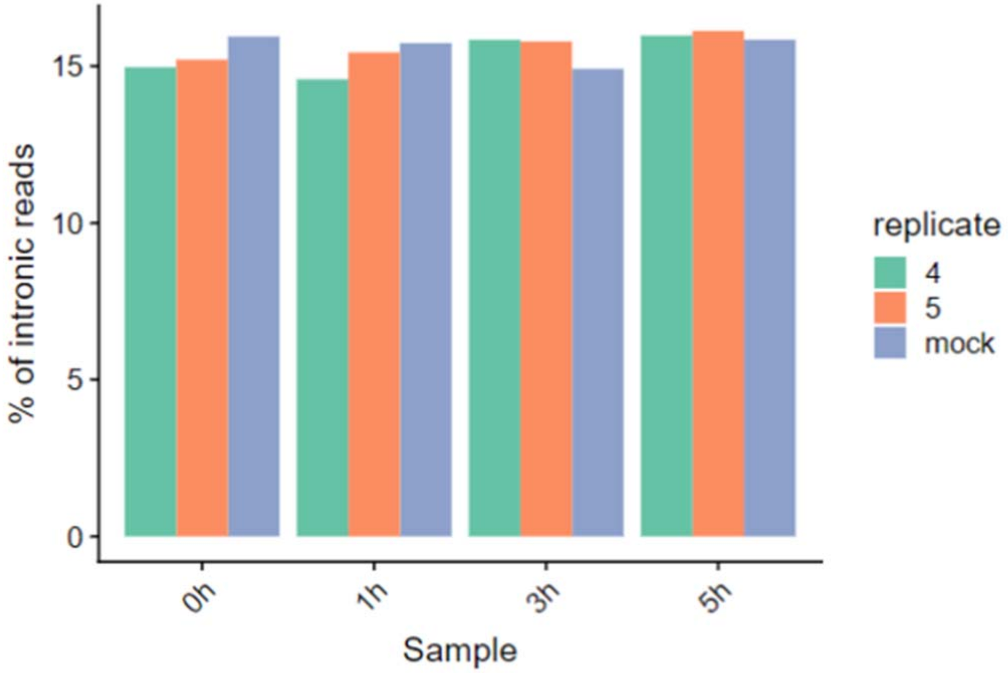


*Figure R1. Intronic reads in RNA-seq data. The bar plots shows per replicate and per time point the fraction read mapping at least partially to an intron by all reads mapping to the human genome.*

**2k. Personally, I find the viral bottlenecks the authors have identified as the most interesting finding. The authors should at least discuss the functions of UL23 and UL50 and how these potentially act as bottlenecks. Maybe an interesting experiment will be to over express these proteins and to test if this affects infection compared to over-expressing of viral genes that the authors predict they do not serve as as major bottlenecks.**
We thank the reviewer for this suggestion. We have performed the experiment, by generating expression plasmids with codon-optimized versions of UL23 and UL50, and, as negative controls, for US and UL33. For all four genes, both N- and C-terminally HA-tagged constructs have been generated, in case the tag at one end would impair the function of the protein. HEK 293 (chemical transfection) and NHDF (Amaxa 4D Nucleofection) cells have been co-transfected with all possible combinations, with empty plasmids and untreated cells as additional negative controls. We observed strong expression (see Fig. R2 below) and transfection efficiencies higher than 50% in both cell types. We could not, however, find an effect of the overexpression on viral transcription as measured by RT-qPCR of 12 viral genes (ranging from US1 to UL33). We mention these attempts only briefly in the discussion of the revised manuscript, but will make

the eight expression plasmids available to other researchers through Addgene to foster research into these directions.
Although negative experiments should be interpreted with caution, we think that this rather points to the hypothesis that the stepwise induction of viral genes is not (only) dependent on viral factors (apart obviously from the well described role of ICP0, ICP4, ICP22 or ICP27), but could also be influenced by the cellular environment.
We have therefore rewritten this section and provided an additional and simpler way of displaying the appearance of viral transcripts (Fig. 2c, Supplementary Fig. 2b). We admit that our previous wording of "bifurcations" and "bottlenecks" was not substantiated enough, but still think that the "set-wise emergence" as we put it now, is supported by the data, and could lead to a more refined temporal categorization of viral genes in addition to the canonical immediate early/early/late categorization. To recapitulate the current definition (as described in the 6th edition of Fields Virology), we have in the color coding in Fig. 2a separated the gamma/gamma1/gamma2 stage and integrated this visual aid into Supplementary Fig. 2b.

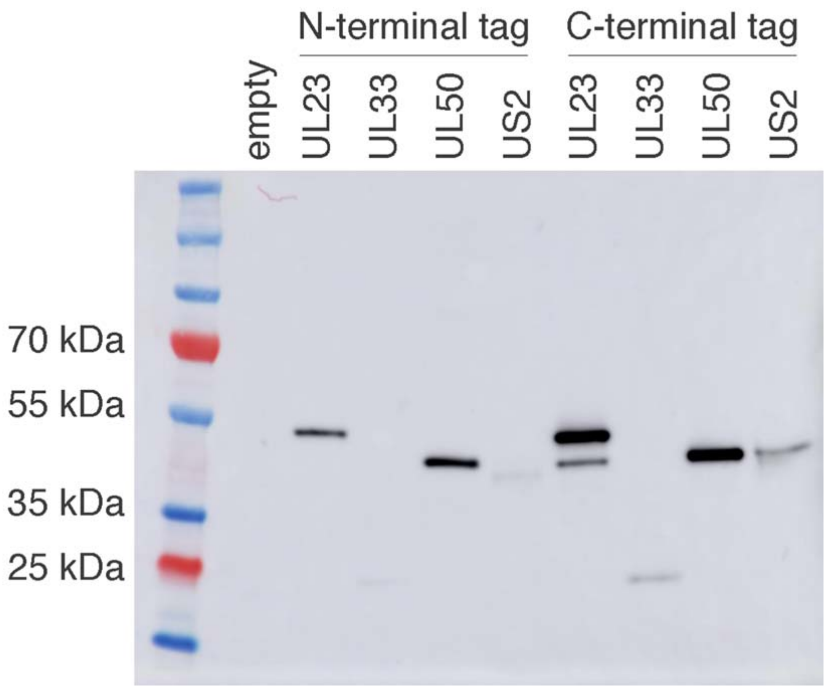


*Figure R2: HEK 293 cells were transfected with expression constructs bearing either UL23, UL33, UL50 or US2 with either an N-terminal or C-terminal HA tag. The expression of proteins was probed using an HA-antibody.*

**2l. In general, it will be of great value if the authors can generate accessible tables that will contain some of the data about viral and cellular gene expression from their single cells (so it could be used as a fruitful ground for future research). For example a table that contain all the viral reads from the single cells. A table that summaries the differential expression of**

**the bulk RNA—seq analysis. A table with the correlation co-efficient of viral genes with host genes etc…**

The GEO record associated with the paper at https://www.ncbi.nlm.nih.gov/geo/query/acc.cgi?acc=GSE123782 contains (in addition to the raw sequencing data) tables with raw counts and normalized counts for the single-cell data, as well as expression values for the bulk RNA-seq. We leave it up to the editor's and reviewer's discretion whether these (huge) tables should be added as Supplementary tables. We agree that a reduced table containing only viral reads and the correlations from Fig. 3 would be useful as Supplementary tables and have added them as Supplementary Table 5 (viral read counts) and Supplementary Table 6 (correlation coefficients).

## Reviewer #3 (Remarks to the Author):

**3a. The authors should validate the finding that NRF2 activation as an antiviral mechanism in a subpopulation of cells using cells harboring an NRF2 reporter. Infect the cells with HSV-1, perform flow cytometry live cell sorting to sort out NRF2 activated cell and corresponding NRF2 inactive cells. Then, the authors should be able to follow and compare those cell populations to see if the NRF2 activated population are indeed less infected with virus.**

We would like to thank the reviewer for this insightful comment, since it allows monitoring the intrinsic homogeneity of the NRF2 activity within the infected cell population. As suggested, we performed such a reporter assay. We transfected a plasmid harboring a constitutionally expressed BFP (for normalization of the transfection efficiency), and a GFP under control of eight times the antioxidant response element (ARE), combined with a minimal promoter (following the scheme of the 8XARE luciferase reporter plasmid in Wang et al. 2006). Upstream of the 8xARE, we have put a polyadenylation signal and a pause site - to reduce RNA polymerase II read-through transcription and therefore background expression of GFP (Fig. 6d). The reporter plasmid reacted to NRF2 stimulation, as expected, and indeed cells with a high NRF2 activity (derived from the ratio of GFP to BFP), were less susceptible to infection than those with low NRF2 activity. The results of this experiment have been added to Fig. 6. Note that we included a data-intrinsic control to this experiment, in which populations gated orthogonal to the NRF2 high/low separated ones showed no difference in susceptibility to infection (Fig. 6h).

The data here is obtained in HEK 293 cells, which we have previously employed as a secondary cell line showing the inhibitory effect of Bardoxolone methyl on the infection. Primary cells such as the primary fibroblasts used for the scRNA-seq are notoriously difficult to transfect, and despite eventually reasonable transfection efficiencies of about 50% using the Amaxa 4D nucleofector system, we observed strong auto-fluorescence in the BFP and mCherry channels (see Fig. R3 below). Of note, we observed on various occasions that infections in HEK 293 cells progress slower than in fibroblasts, which explains the relatively large amount of cells that have still very low levels of mCherry in Fig. 6.

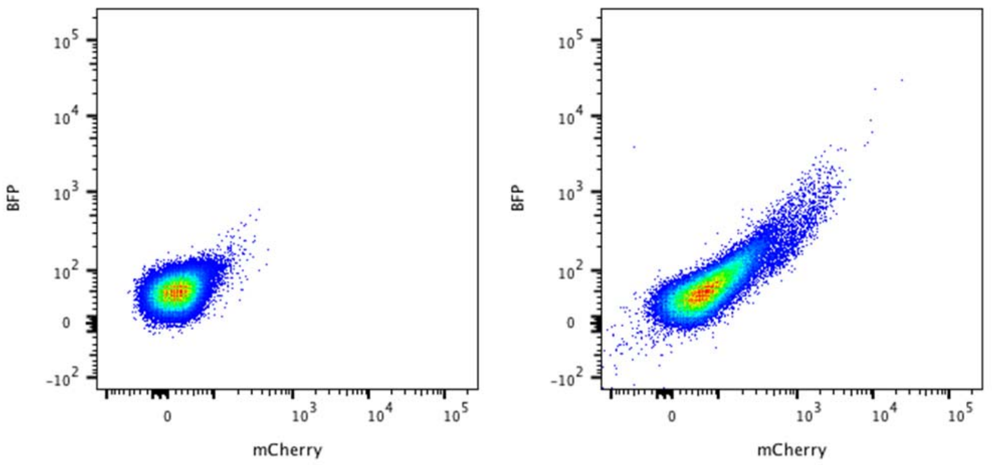


*Figure R3: HEK 293 cells (left) and NHDF cells (right) were transfected with an empty plasmid and subjected to FACS analysis. Shown are the signal in the mCherry channel (horizontal axis) and the BFP channel (vertical axis).*

**3b. In addition to Bardoxolone methyl, the authors should use additional NRF2 inducers coupled with the use of NRF2 knockout cells to cross validate their findings. These experiments are important to make sure that the observed antivirus effect is not specific to bardoxolone methyl and is NRF2 dependent.**

We added experiments using a second NRF2 activator, Sulforaphane, (described in Fig. 6). Sulforaphane was previously shown to activate NRF2 in the low micromolar range (Fujie et al. 2016), and at these concentrations also reduced HSV-1 virus production. We agree that NRF2 knockdown would further substantiate our findings. However, in a recent paper, which we have previously included in the discussion (Olagnier et al. 2018), knockdown of NRF2 led to an induction of the HSV-1 restricting factors IFI16 and particularly STING. This indicates that experiments including strong perturbations of the system for longer time periods could obfuscate interpretations due to uncontrollable indirect effects. In light of this, we would like to emphasize that two out of three of our lines of evidence (scRNA-seq and FACS, with NRF2 agonist treatment being the third) for a restriction of HSV-1 infection come from perturbation-free experiments, in which the intrinsic heterogeneity of NRF2 activity was measured and related to HSV-1 infection on the level of single cells. In our opinion, this approach is more apt since indirect/side effects are less likely.

**3c. Please include a data transformation section in the materials and methods section. Please demonstrate homoscedasticity of the transformed data used to calculate linear regression correlation coefficient.**

We followed the reviewer's suggestion and added a description of data transformation to the methods section.

To demonstrate that the distributional properties of single cell data did not bias the correlation statistics, we have repeated the correlation analysis in Fig. 3b (Y axis) with variance stabilized (VST) data (sctransform: Hafemeister et al. 2019). sctransform uses negative binomial regression to remove any apparent relationship between mean and variance, making the data homoscedastic.
Supplementary Fig. 1d shows correlation of gene expression across cells, with the viral load, before (X axis), and after the variance stabilizing transformation (Y axis). Transformed correlation statistics do not differ significantly from the non-transformed statistic, demonstrating that the conclusions based on the correlation statistics were unbiased.

**3d. Figure 2a is not clear. Please describe the meaning of the horizontal dotted line. Is this a threshold by which subsequent genes were selected?**
**3e. Figure 2b: Please explain why was UL24 discounted, considering it passed the dotted line threshold.**
The cutoff and therefore the number of genes that are analyzed in the main figure (additional ones are in Supplementary Fig. 2) is somewhat arbitrary, in order to keep the main figure simple. We deleted the dashed line since it is only confusing.

**3f. Figure S2A should be moved to main Figure 2 to make it easier for readers to understand the definition of "HSV-1 high".**
Supplementary Fig. 2a has been moved to Fig. 2 as suggested.

**3g. Figure 2b and Figure S2b are confusing. Is Figure S2B necessary?**
We have extended both figures.b. 1b is particularly important for the visualization of the cells at the very beginning for which either US1 or UL54 was detected. In addition, these figures present a better visualization temporal dynamics than the other panels. However we do not insist on Supplementary Fig. 2 and leave it up to the editor's and reviewer's discretion whether to omit or include it.

**3h. Line 142, shouldn't that be referring to Fig. S2A?**
We were actually referring to Fig. 1e, however this sentence is not present any more.

**3i. Figure 3d: y-axis title is misleading. For example "0.08 viral DNA in supernatant" does not make much sense. Please consider revising that y-axis title to PFU/ml.**
We have changed the y-axis title to "relative amount of viral DNA in supernatant" and added here and for Fig. 6 plaque assays of the supernatants.

**3j. Watch spelling: e.g., line 33 "transcrips" = "transcripts"**
This spelling mistake and others have been corrected.

**3k. Line 96: what is nUMI defined as? Acronyms should be spelled out in the main text.**
We added an explanation to the first paragraph of the result section.

**3l. Figure 2: “HSV-1 high” definition should be supplied in main text and/or figure legend, and not in supplemental. Figure needs to be able to stand on its own without referencing supplemental information to interpret the figure.**
Supplementary Fig. 2a, with the definition of "HSV-1 high", has been moved to Fig. 2 as suggested.

**3m. Line 126-127: recommend changing “start mostly around” to “start in regions flanking the internal repeat regions”.**
This part was changed as suggested.

**3n. Figure 2a: Please label the bars in the IRL and IRS regions with their gene(s)**
The missing legends were added to the figure.

**3o. Figure 2b, legend: “experiment two” is an unclear definition. Also, “HSV-1 high” is not defined in main text results section, and the figure should stand alone without referring to supplemental figures. Supp Fig 2 seems particularly important and perhaps should be moved to main text.**
The "experiment two" was a leftover from some old figure versions and was deleted. Supplementary Fig. 2a has been moved to the main figure as suggested.

**3p. Temper the wording in lines 139-141: while I agree with the authors’ conclusions that generally UL1 and UL54 are transcribed prior to UL34 and UL50, there are notable subpopulations that expression UL34 and/or UL50 in the absence of UL1 and UL54. So it is not universal that US1 and UL54 were transcribed before 23 and 50 (line 140).**
This section has been rewritten (see answer to reviewer comment 2k above) and the results have been described in a different and more cautious way.

**3q. Line 142: “as shown above” in the main text should be “as shown in Fig. 2a”?**
**3r. Line 142: I do not see a bimodal distribution in Fig. 2a, and Fig. 2a; is this actually referring to Fig 1e?**
This is indeed referring to Fig. 1e, however this sentence is now deleted.

**3s. Lines 683-684: refers to Figures 3 and 4, not 4 and 5?**
The references were corrected.

**3t. Gene names should be italicized**
Gene names are now italicized

REVIEWERS' COMMENTS:

Reviewer #1 (Remarks to the Author):

In the reply to my comments, the authors state that their mail goal is to "provide a detailed picture of temporal events and cellular heterogeneity in HSV-1 infected cells". I agree that this is valuable and that such analysis will always be, in a sense, descriptive in nature. However, this does not seem to be reflected in the wording of the headline. The headline indicates that the authors have very strong data that NRF2 controls an antiviral program. However, there is no attempt to test if the effect of SFN an Bardoxolone is NRF2 dependent. The consequence of this is that the headline is not sufficiently supported by the data. Much could be gained if the headline reflects better the nature of the study, namely that it describes (and pretty well I think), the sequence of transcriptional events that occur during infection.

Reviewer #2 (Remarks to the Author):

I have no additional comments

Reviewer #3 (Remarks to the Author):

The authors have done a fantastic job in addressing reviewers comments and the manuscript is very much improved.

One minor suggestion:
The modest change seen in the ARE-reporter-FACS assays is likely due to the stability of the GFP protein. The results could be further improved by using a destabilized variant of GFP. This can be done by fusing a PEST degron to the c-terminal of the GFP protein as shown in Kerins MJ et al. 2019. Mol Cell Biol. 2019 Jun 13;39(13) PMID: 31010806
